# Patient-Reported Health Status and Clinical Outcomes After CTO-PCI Versus Optimal Medical Therapy: A 12-Month Retrospective Observational Cohort Analysis

**DOI:** 10.3390/jcm15145668

**Published:** 2026-07-20

**Authors:** Velina Doktorova, Georgi Goranov, Petar Nikolov, Mariyan Marinov

**Affiliations:** 1 First Department of Internal Diseases, Section of Cardiology, Medical University of Plovdiv, 4000 Plovdiv, Bulgaria; 2 Department of Interventional Cardiology, UMHAT “Sveti Georgi”, 4000 Plovdiv, Bulgaria; 3 Department of Cardiology, Section of Interventional Cardiology, MHAT “Sveti Ivan Rilski”, 4000 Plovdiv, Bulgaria

**Keywords:** chronic total occlusion, CTO-PCI, optimal medical therapy, Seattle Angina Questionnaire, patient-reported outcomes, angina frequency, health status, quality of life, cardiovascular rehospitalization, chronic coronary syndrome, retrospective cohort study

## Abstract

**Background/Objectives:** Chronic total coronary occlusion (CTO) is frequently associated with persistent angina, functional limitation, and impaired health-related quality of life. Although CTO percutaneous coronary intervention (CTO-PCI) has improved technically, its clinical value is best assessed through patient-reported outcomes (PROs) and patient-centered clinical endpoints. This study evaluated the association between CTO-PCI plus optimal medical therapy (OMT) and 12-month patient-reported and clinical outcomes compared with OMT alone. **Methods:** This single-center, non-randomized retrospective observational cohort analysis included 251 patients with chronic coronary syndrome and angiographically confirmed CTO. Patients were managed with either a CTO-PCI + OMT strategy (*n* = 153) or OMT alone (*n* = 98). The analysis used routinely collected clinical, angiographic, echocardiographic, procedural, and follow-up data, complemented by patient-reported Seattle Angina Questionnaire (SAQ) data obtained after written informed consent. The overall SAQ assessment was considered the primary patient-reported framework, and SAQ Angina Frequency was defined as the principal symptom-specific domain for adjusted comparative analysis. Propensity-score overlap weighting was used as a sensitivity analysis to address measured baseline imbalance and treatment-selection bias. Secondary outcomes included Canadian Cardiovascular Society angina class, left ventricular ejection fraction, cumulative 12-month cardiovascular rehospitalization, and survival. **Results:** The complete-case SAQ population included 217 patients: 136 in the CTO-PCI + OMT group and 81 in the OMT group. Thirty-four patients lacked complete 12-month SAQ data: 24 died before SAQ reassessment, and 10 were lost to SAQ follow-up. SAQ Angina Frequency improved in both groups, from 70.81 ± 17.97 to 92.35 ± 12.37 in the CTO-PCI + OMT group and from 63.09 ± 20.10 to 83.09 ± 15.14 in the OMT group. In the multivariable ANCOVA model, CTO-PCI + OMT was associated with higher 12-month SAQ Angina Frequency compared with OMT alone (β = 7.07 points; 95% CI, 3.36–10.79; *p* < 0.001). This finding remained consistent in the propensity-score overlap-weighted sensitivity analysis adjusted for baseline SAQ Angina Frequency (β = 6.55 points; 95% CI, 2.39–10.70; *p* = 0.002). Cardiovascular rehospitalization was observed in 11 of 153 patients (7.2%) in the CTO-PCI + OMT group and in 19 of 98 patients (19.4%) in the OMT group, corresponding to lower odds of cumulative 12-month rehospitalization (OR = 0.32; 95% CI, 0.15–0.71; *p* = 0.005). The rehospitalization endpoint was analyzed as a cumulative binary outcome rather than as a time-to-event outcome. Overall survival estimates were 92.0% and 87.4%, respectively, with 12 deaths in each group (log-rank *p* = 0.225). Because only 24 deaths were recorded, the study was underpowered to evaluate mortality differences. **Conclusions:** In selected patients with chronic coronary syndrome and CTO, CTO-PCI + OMT was associated with greater improvement in disease-specific health status, particularly SAQ Angina Frequency, compared with OMT alone. CTO-PCI + OMT was also associated with lower cumulative 12-month cardiovascular rehospitalization, although this secondary finding should be interpreted cautiously because of the non-randomized retrospective design, modest event numbers, lack of time-to-event rehospitalization data, and potential expectation- and care-related biases. Survival analyses were underpowered and should not be interpreted as evidence of either prognostic benefit or absence of benefit. These findings support consideration of CTO-PCI as a patient-centered therapeutic option in carefully selected symptomatic patients, while acknowledging residual confounding by indication and the open-label study design.

## 1. Introduction

Chronic total occlusion (CTO) of a coronary artery, commonly defined as complete interruption of antegrade coronary flow with Thrombolysis in Myocardial Infarction (TIMI) grade 0 for an estimated or documented duration of at least three months, is a frequent finding among patients undergoing coronary angiography for obstructive coronary artery disease [1,2,3]. Beyond its angiographic definition, CTO represents a clinically relevant phenotype of chronic coronary syndrome, often associated with persistent angina, inducible ischemia, impaired functional capacity, and reduced health-related quality of life [2,3,4]. Over the last decade, dedicated equipment, structured crossing algorithms, hybrid antegrade and retrograde strategies, and increasing operator experience have substantially improved the technical success of CTO percutaneous coronary intervention (CTO-PCI), particularly in specialized centers [3,4]. Nevertheless, the central clinical question is no longer limited to whether an occluded vessel can be recanalized, but rather which patients are most likely to derive meaningful symptomatic, functional, and disease-specific health-status benefit from revascularization.

Despite these advances, the role of CTO-PCI remains debated. Compared with PCI of non-occlusive coronary lesions, CTO-PCI is technically more demanding, resource-intensive, and associated with longer procedure time, greater contrast and radiation exposure, and a non-negligible risk of periprocedural complications [3,4]. In addition, randomized evidence has not consistently demonstrated a reduction in hard clinical endpoints such as death or myocardial infarction. The DECISION-CTO trial did not show superiority of routine CTO-PCI plus optimal medical therapy (OMT) over OMT alone for major adverse cardiovascular outcomes, whereas EUROCTO demonstrated significant improvements in angina frequency and quality of life in patients randomized to CTO-PCI [5,6]. IMPACTOR-CTO further supported the potential of CTO revascularization to reduce ischemic burden and improve functional and quality-of-life measures in selected patients [7]. More recent syntheses have reinforced this nuanced interpretation: CTO-PCI may improve patient-centered outcomes, while its prognostic effect remains uncertain and hypothesis-generating [8,9,10]. Importantly, the 2026 pooled analysis of EUROCTO and DECISION-CTO showed superior improvement in Seattle Angina Questionnaire (SAQ) angina frequency, quality of life, and summary scores with PCI compared with OMT, without a signal of excess harm regarding clinical endpoints [10]. In parallel, the sham-controlled ORBITA-CTO trial provided contemporary evidence that CTO-PCI can reduce angina burden beyond placebo effects in carefully selected patients with symptomatic single-vessel CTO [11]. These findings underscore both the clinical relevance of symptom relief and the importance of cautious interpretation of unblinded symptom-driven outcomes.

In this context, patient-reported outcomes (PROs) have become essential for evaluating the clinical value of symptom-directed cardiovascular interventions. PROs capture information on a patient’s health status directly from the patient, without interpretation by clinicians or other observers, and are particularly important when treatment benefit is expected to involve symptoms, functional capacity, treatment satisfaction, or health-related quality of life [12,13]. In chronic coronary syndromes, where mortality or myocardial infarction may be infrequent during short- or medium-term follow-up, PROs provide a structured and patient-centered way to quantify clinically meaningful treatment effects that may not be fully reflected by angiographic success, physician-assessed angina class, or traditional event-based endpoints.

The Seattle Angina Questionnaire is a disease-specific, patient-reported instrument developed to quantify five clinically important domains of coronary artery disease: physical limitation, angina stability, angina frequency, treatment satisfaction, and disease-specific quality of life [14]. Its validity, reproducibility, and responsiveness to clinical change have been demonstrated in patients with coronary artery disease, and SAQ-based health-status assessment has been incorporated into major trials of chronic coronary syndromes [14,15,16]. For CTO patients in particular, SAQ offers a standardized way to capture outcomes that are directly meaningful to patients, including the frequency of anginal symptoms, their impact on daily activities, and the perceived effect of treatment on quality of life.

However, important gaps remain in the evidence base. Randomized trials provide high internal validity but are often limited by slow recruitment, selective inclusion criteria, crossover, and underpowering for hard clinical endpoints. Conversely, observational studies and registries reflect real-world practice but are vulnerable to selection bias and frequently focus on procedural success or survival rather than structured patient-reported health status. Therefore, additional real-world observational analyses with structured patient-reported outcome assessment and explicit attention to treatment-selection bias data are needed to evaluate how the choice of therapeutic strategy—CTO-PCI plus OMT or OMT alone—is associated not only with survival, but also with angina burden, functional limitation, left ventricular function, rehospitalization, and quality of life during follow-up.

The aim of the present study was to evaluate the association between treatment strategy and 12-month patient-reported and clinical outcomes in patients with chronic coronary syndrome and angiographically confirmed CTO. We specifically compared CTO-PCI plus OMT with OMT alone in a single-center, non-randomized retrospective observational cohort analysis of routinely collected clinical, angiographic, echocardiographic, procedural, and follow-up data, complemented by patient-reported SAQ data obtained after written informed consent. The overall SAQ assessment was considered the primary patient-reported framework, with SAQ Angina Frequency defined as the principal symptom-specific domain for adjusted comparative analysis. Secondary outcomes included CCS angina class, LVEF, cumulative 12-month cardiovascular rehospitalization, and survival. All comparative analyses were interpreted as associations rather than causal treatment effects.

## 2. Materials and Methods

### 2.1. Study Design and Setting

This was a single-center, non-randomized retrospective observational cohort analysis conducted at the Department of Interventional Cardiology, St. George University Hospital, Plovdiv, Bulgaria. The analysis used routinely collected clinical, angiographic, echocardiographic, procedural, and follow-up data from adult patients with chronic coronary syndrome and angiographically confirmed chronic total coronary occlusion who were managed in routine clinical practice between June 2018 and June 2024. These data were complemented by patient-reported Seattle Angina Questionnaire (SAQ) assessments obtained after written informed consent for the health-status assessment component.

A total of 251 adult patients were included. The analysis compared two treatment strategies used in routine clinical practice: an interventional strategy consisting of attempted chronic total occlusion percutaneous coronary intervention in addition to optimal medical therapy (CTO-PCI + OMT; *n* = 153), and a conservative strategy consisting of OMT alone (*n* = 98).

Treatment allocation was not randomized, was not protocol-mandated, and did not represent assignment within an interventional clinical trial. The therapeutic strategy was selected as part of routine clinical care after clinical, angiographic and, when appropriate, functional assessment, taking into account symptom burden, evidence of ischemia and/or myocardial viability, anatomical feasibility of CTO-PCI, expected procedural complexity and risk, comorbidities, operator judgment, and patient preference. Patients in the CTO-PCI + OMT group were analyzed according to the selected interventional treatment strategy, regardless of final procedural success.

Patients in the OMT group did not undergo CTO-PCI at baseline because of informed refusal, an unfavorable individualized expected risk–benefit profile, or high anticipated procedural complexity or risk as assessed by an experienced operator. These patients were distinct from patients excluded from the analysis because CTO anatomy was considered unsuitable for any reasonable attempt at safe and effective PCI. In the OMT group, CTO-PCI was considered technically conceivable but was not selected in routine clinical practice because the expected symptomatic or clinical benefit was judged insufficient to justify the anticipated procedural complexity or risk, or because the patient declined intervention.

The index date was defined as the date of treatment-strategy selection. Baseline evaluation corresponded to the clinical, angiographic, echocardiographic, and patient-reported assessment available at or immediately before this index date. Follow-up was assessed for 12 months after treatment-strategy selection and included angina status, left ventricular function, cardiovascular and cerebrovascular events, repeat revascularization, cardiovascular rehospitalization, and survival. All patients received OMT according to contemporary guideline-based clinical practice and individualized physician judgment.

The study was open-label. Patients and treating physicians were aware of the treatment strategy selected in routine clinical practice, and no sham-control procedure was performed. Therefore, patient-reported and symptom-driven outcomes were interpreted in the context of possible expectation effects, treatment-perception bias, physician perception, and follow-up behavior.

### 2.2. Study Population and Eligibility Criteria

Eligible patients were adults aged ≥18 years with chronic coronary syndrome and angiographically confirmed chronic total coronary occlusion of at least one epicardial coronary artery who were managed during the study period and had available baseline clinical, angiographic, echocardiographic, procedural where applicable, and follow-up data sufficient for the present retrospective observational analysis. CTO was defined as complete interruption of antegrade coronary flow through the target lesion, corresponding to Thrombolysis in Myocardial Infarction (TIMI) grade 0 flow, with an estimated or documented duration of at least three months. The duration of the occlusion was determined on the basis of previous angiographic findings, clinical history, prior myocardial infarction in the corresponding territory, or a stable pattern of symptoms consistent with chronic coronary occlusion.

Patients were considered eligible if they had angina or ischemic equivalents consistent with chronic coronary syndrome, persistent despite optimal medical therapy, and/or objective evidence of ischemia in the myocardial territory supplied by the occluded vessel. Evidence supporting viability in the CTO-related territory was required as part of routine clinical decision-making. This assessment was based on resting echocardiographic wall-motion evaluation and, when clinically indicated, additional functional testing. In patients with regional wall-motion abnormalities, myocardial viability was further assessed by pharmacological stress echocardiography when clinically indicated. All patients underwent clinical and anatomical assessment of CTO-PCI feasibility by an experienced interventional cardiologist. In patients with multivessel coronary artery disease, significant non-occlusive coronary stenoses were treated or planned for treatment before final assessment of the target CTO strategy.

The available dataset systematically captured whether dobutamine stress echocardiography was performed, but did not uniformly capture quantitative ischemic burden, quantitative viability extent, or results from advanced imaging modalities such as stress cardiac magnetic resonance, nuclear perfusion imaging, or positron-emission tomography.

Patients were excluded if they had contraindications to dual antiplatelet therapy, including active bleeding, hemorrhagic diathesis, or high risk of major bleeding complications; pregnancy or breastfeeding; severe non-cardiac comorbidity with an expected life expectancy of less than one year; clinical or anatomical indications for surgical revascularization; planned major surgery within the following three months requiring interruption of antithrombotic therapy; target CTO anatomy considered unsuitable for any reasonable attempt at safe and effective PCI, such that CTO-PCI was not considered a clinically appropriate therapeutic option; absence of objective evidence of ischemia or viable myocardium in the CTO-related territory; or insufficient baseline or follow-up information to classify treatment strategy and ascertain clinical outcomes.

The criterion of anatomy unsuitable for PCI referred to patients in whom CTO-PCI was considered inappropriate as a therapeutic option because the anatomical likelihood of safe and effective recanalization was judged prohibitively low or the procedural risk unacceptably high. This exclusion criterion differed from inclusion in the OMT group, where CTO-PCI was technically conceivable but was not selected in routine clinical practice because the individualized expected risk–benefit balance was unfavorable, anticipated procedural complexity or risk was high relative to expected benefit, or the patient declined intervention.

Baseline demographic characteristics, cardiovascular risk factors, comorbidities, previous cardiovascular history, angina status, left ventricular ejection fraction, angiographic characteristics, CTO complexity, and baseline medical therapy were recorded at the index assessment corresponding to treatment-strategy selection. Patient-reported health status and disease-specific quality of life were assessed using the Seattle Angina Questionnaire at baseline and at 12-month follow-up. Patients who died before 12-month SAQ reassessment or were lost to SAQ follow-up were retained in the overall clinical cohort when clinical follow-up status was ascertainable, but were excluded from complete-case SAQ analyses as described below.

### 2.3. Treatment Strategy Groups

Patients were categorized into two treatment-strategy groups according to the therapeutic approach selected in routine clinical practice at the index assessment. The interventional group consisted of patients in whom a CTO-PCI strategy in addition to optimal medical therapy was undertaken (CTO-PCI + OMT group; *n* = 153). The conservative group consisted of patients managed with optimal medical therapy alone without attempted CTO-PCI at baseline (OMT group; *n* = 98).

Treatment strategy was not randomly assigned, was not protocol-mandated, and did not represent allocation by a research intervention. The decision to proceed with CTO-PCI or to continue OMT alone was made as part of routine clinical care after integrated clinical, angiographic and, when appropriate, functional evaluation. The decision-making process considered symptom burden, objective evidence of ischemia and/or viable myocardium, anatomical feasibility for CTO-PCI, anticipated procedural complexity and risk, comorbidities, physician and operator judgment, and patient preference.

In the CTO-PCI + OMT group, an attempt was made to recanalize the target CTO, and patients were analyzed according to the selected interventional treatment strategy regardless of final procedural success. This strategy-based classification was retained for all primary comparative analyses because the clinical decision in routine practice is whether to pursue a CTO-PCI + OMT strategy before procedural success is known. Procedural success and periprocedural complications were analyzed separately within the CTO-PCI + OMT group.

In the OMT group, CTO-PCI was not attempted at baseline despite technically conceivable revascularization because the patient declined intervention, the individualized expected symptomatic or clinical benefit was judged insufficient to justify the anticipated procedural complexity or risk, or the treating operator considered the overall risk–benefit profile unfavorable in routine clinical practice. These patients were distinct from excluded patients in whom CTO anatomy was considered unsuitable for any reasonable attempt at safe and effective PCI.

All patients in both groups received OMT according to contemporary guideline-based clinical practice and individualized physician judgment. OMT was considered an active treatment strategy rather than absence of intervention and could be initiated, intensified, discontinued, or modified during follow-up according to symptoms, tolerance, clinical status, procedural findings where applicable, ischemic and bleeding risk, and physician judgment.

### 2.4. Baseline Clinical, Imaging and Functional Assessment

At the index clinical assessment corresponding to treatment-strategy selection, all patients underwent routine clinical evaluation according to departmental practice. Demographic characteristics, cardiovascular risk factors, comorbidities, previous myocardial infarction, previous percutaneous or surgical coronary revascularization, smoking status, and current cardiovascular medications were recorded from medical records and clinical documentation. Clinical presentation was assessed according to angina symptoms, ischemic equivalents, and Canadian Cardiovascular Society (CCS) angina class. A standard 12-lead electrocardiogram was performed in all patients, and routine laboratory parameters, including lipid profile and renal function, were analyzed when available.

Transthoracic echocardiography was performed at baseline and at 12-month follow-up. The main echocardiographic parameter of interest was left ventricular ejection fraction (LVEF), which was used for baseline characterization and for assessment of functional change during follow-up. Regional wall motion was evaluated using a segmental model, with particular attention to the myocardial territory supplied by the occluded vessel. LVEF was interpreted as a global echocardiographic estimate of systolic function and was analyzed as a secondary exploratory outcome. Because small differences in echocardiographic LVEF may fall within expected measurement variability, LVEF findings were interpreted cautiously and were not considered mechanistic evidence of improved ventricular function. Systematic blinding of echocardiographic readers to treatment strategy was not documented.

Dobutamine stress echocardiography was performed when clinically indicated, particularly in patients with regional wall-motion abnormalities or when additional assessment of contractile reserve and inducible ischemia was needed for treatment-strategy selection. This modality allowed assessment of contractile reserve as a marker of viability and, at higher stress levels, evaluation of inducible ischemia. The performance of dobutamine stress echocardiography was recorded as a binary variable. Quantitative ischemic burden and quantitative viability extent were not uniformly available in the dataset and were therefore not included as comparative variables.

Coronary angiography was reviewed to confirm the presence of CTO, identify the target vessel, assess the number of diseased coronary vessels, and estimate CTO lesion complexity. The target CTO vessel was categorized as the right coronary artery, left anterior descending artery, left circumflex artery, or left main coronary artery, where applicable. CTO complexity was assessed using the J-CTO score and categorized according to established difficulty grades. Anatomical feasibility for CTO-PCI was evaluated by an experienced interventional cardiologist, taking into account lesion morphology, expected crossing difficulty, distal vessel quality, collateral circulation, and anticipated procedural complexity and risk.

Anatomical assessment was used both to identify patients in whom CTO-PCI was a reasonable potential option and to exclude patients in whom the anatomy was considered unsuitable for any reasonable attempt at safe and effective PCI. Among patients retained in the analytic cohort, anticipated procedural complexity and risk could still influence selection of CTO-PCI + OMT versus OMT alone.

In patients with multivessel coronary artery disease, significant non-occlusive coronary stenoses were treated or planned for treatment before final assessment of the target CTO strategy. This approach was used to ensure that symptoms, ischemia, and patient-reported health status were interpreted in relation to the CTO lesion and the overall coronary anatomy.

### 2.5. CTO-PCI Procedure and Optimal Medical Therapy

In patients selected for an interventional strategy, attempted CTO-PCI was performed by experienced operators according to contemporary interventional practice. The choice of procedural approach was left to the operator’s discretion and was based on coronary anatomy, lesion complexity, proximal and distal cap morphology, collateral circulation, expected technical feasibility, and procedural risk. Antegrade wire escalation, antegrade dissection/re-entry, retrograde techniques, and hybrid crossing strategies were used when appropriate according to the anatomical characteristics of the target CTO.

Technical success was defined angiographically as successful recanalization of the target CTO with restoration of TIMI grade 3 antegrade flow and residual stenosis < 30% in the treated segment. Procedural and angiographic variables recorded in the available dataset included technical success, post-procedural TIMI flow, residual stenosis after PCI, and selected in-hospital periprocedural complications. Periprocedural complications included procedural myocardial infarction, coronary perforation, cardiac tamponade, stroke, periprocedural death, and urgent coronary artery bypass grafting. Procedural success and periprocedural complications were analyzed separately within the CTO-PCI + OMT group.

Detailed procedural metrics, including access site, antegrade versus retrograde crossing strategy, use of dissection/re-entry, contrast volume, fluoroscopy time, radiation dose, number and type of implanted stents, and total procedural duration, were not uniformly available in the dataset and therefore could not be analyzed quantitatively.

All patients received optimal medical therapy according to contemporary guideline-based clinical practice and individualized physician judgment. OMT included antianginal therapy, antiplatelet therapy when indicated, lipid-lowering therapy, and pharmacological management of hypertension, heart rate, diabetes, and other cardiovascular risk factors. Antianginal therapy was individualized according to symptom burden, blood pressure, heart rate, comorbidities, and treatment tolerance. OMT was considered an active treatment strategy rather than absence of intervention and could be initiated, intensified, discontinued, or modified during follow-up according to symptoms, tolerance, clinical status, procedural findings where applicable, ischemic and bleeding risk, and physician judgment.

Medical therapy was recorded at baseline and at 12-month follow-up according to prespecified medication classes. These included aspirin, P2Y12 receptor inhibitors, beta-blockers, angiotensin-converting enzyme inhibitors, angiotensin II receptor blockers, calcium-channel blockers, statins, other lipid-lowering drugs, nitrates, trimetazidine, ranolazine, and ivabradine. Medication use was assessed as present or absent for each class.

Patients scheduled for CTO-PCI were prepared according to standard interventional cardiology protocols. Before the procedure, patients received antiplatelet therapy, including aspirin and a P2Y12 receptor inhibitor, as clinically indicated. During CTO-PCI, intravenous unfractionated heparin was administered, with dosing adjusted to maintain activated clotting time within the therapeutic range appropriate for complex PCI. After CTO-PCI, antiplatelet therapy and other cardiovascular medications were continued according to procedural findings, stent implantation, bleeding risk, ischemic risk, and guideline-based clinical practice. Dual antiplatelet therapy (DAPT) duration and modification were determined according to procedural findings, stent implantation, ischemic risk, bleeding risk, tolerance, and physician judgment. Bleeding events during DAPT were not systematically adjudicated according to Bleeding Academic Research Consortium (BARC) criteria in the available dataset. Therefore, formal BARC-classified bleeding outcomes were not included as prespecified comparative endpoints.

Patients in the OMT group were treated conservatively without attempted CTO-PCI at baseline. Medical therapy could be initiated, intensified, discontinued, or modified during follow-up according to symptoms, tolerance, clinical status, risk-factor control, and physician judgment. Thus, OMT alone represented an active conservative treatment strategy rather than absence of therapy.

### 2.6. Outcomes and Follow-Up

The primary patient-reported assessment was the change in disease-specific health status from baseline to 12-month follow-up, assessed using the Seattle Angina Questionnaire (SAQ). The SAQ was used as a disease-specific patient-reported outcome measure designed to quantify symptom burden, functional limitation, treatment satisfaction, and quality of life in patients with coronary artery disease. Completion of the SAQ was not part of standard routine clinical practice in the department; therefore, SAQ assessments were obtained only after written informed consent for this patient-reported health-status component.

The following SAQ domains were analyzed: Physical Limitation, Angina Stability, Angina Frequency, Treatment Satisfaction, and Quality of Life. Each domain score was transformed to a 0–100 scale, with higher scores indicating better health status, less frequent angina, lower functional limitation, greater treatment satisfaction, and better disease-specific quality of life. The overall SAQ assessment was considered the primary patient-reported framework for evaluating disease-specific health status. Among the SAQ domains, Angina Frequency was defined as the principal symptom-specific domain for adjusted comparative analysis because it directly reflects anginal symptom frequency and the need for symptom-relieving medication. Analyses of the other SAQ domains were interpreted as secondary and exploratory.

SAQ scores were assessed at baseline and at 12 months. For each SAQ domain, baseline score, 12-month score, absolute change from baseline, and between-group difference in change were evaluated. Clinically meaningful improvement was assessed using commonly applied SAQ interpretive thresholds, with an increase of approximately ≥5 points considered small but clinically noticeable, ≥10 points considered moderate, and ≥20 points considered large improvement.

Secondary clinical outcomes included change in angina severity according to the Canadian Cardiovascular Society (CCS) classification, change in left ventricular ejection fraction (LVEF), major adverse cardiac and cerebrovascular events (MACCE), cumulative cardiovascular rehospitalization during 12-month follow-up, and survival. MACCE was defined as a composite of all-cause death, myocardial infarction, stroke, and repeat revascularization. LVEF was analyzed as a secondary exploratory outcome and was interpreted cautiously because small echocardiographic differences may fall within expected measurement variability.

Cardiovascular rehospitalization was defined as unplanned hospitalization due to unstable angina or decompensated heart failure during the 12-month follow-up period. Because exact dates of cardiovascular rehospitalization were not consistently available for all patients, this endpoint was analyzed as a cumulative 12-month binary outcome rather than as a time-to-event outcome.

For patients in the CTO-PCI + OMT group, procedural outcomes were additionally evaluated. These included technical success, post-procedural TIMI flow, residual stenosis after PCI, and selected periprocedural complications recorded during the index hospitalization. Technical success was defined as successful recanalization of the target CTO with restoration of TIMI grade 3 antegrade flow and residual stenosis < 30% in the treated segment. Periprocedural complications included procedural myocardial infarction, coronary perforation, cardiac tamponade, stroke, periprocedural death, and urgent coronary artery bypass grafting. Bleeding events during DAPT were not systematically adjudicated according to BARC criteria in the available dataset and were therefore not analyzed as formal comparative safety endpoints.

Follow-up was assessed 12 months after treatment-strategy selection through outpatient visits, hospital records, and/or telephone contact. Follow-up assessment included SAQ, CCS angina class, echocardiographic assessment of LVEF, occurrence of cardiovascular and cerebrovascular events, repeat revascularization, cardiovascular rehospitalization, and vital status. Clinical event data were obtained from hospital records, follow-up visits, and patient contact when appropriate. Because this was a retrospective observational analysis, all clinical outcomes were interpreted as secondary or exploratory in relation to the primary patient-reported objective of the study.

Because the study was open-label and did not include a sham-control procedure, SAQ, CCS class, and cardiovascular rehospitalization were interpreted in the context of possible patient expectation effects, treatment perception, physician perception, follow-up behavior, and thresholds for hospital admission.

### 2.7. Statistical Analysis

Statistical analyses were performed using IBM SPSS Statistics version 27.0 (IBM Corp., Armonk, NY, USA) and MedCalc version 19.6.3 (MedCalc Software Ltd., Ostend, Belgium). Additional propensity-score weighting analyses and weighted regression models with robust standard errors were performed using Python version 3.13.5 (Python Software Foundation, Beaverton, OR, USA), with statsmodels version 0.14.6 and scikit-learn version 1.8.0. A two-sided *p*-value < 0.05 was considered statistically significant for the principal analyses. For secondary and exploratory analyses, *p*-values were interpreted as nominal because no formal adjustment for multiple comparisons was applied.

Continuous variables were assessed for distributional normality using the Kolmogorov–Smirnov and Shapiro–Wilk tests, together with graphical inspection of histograms when appropriate. Normally distributed continuous variables were presented as mean ± standard deviation, whereas non-normally distributed variables were presented as median and interquartile range. Categorical variables were summarized as absolute numbers and percentages.

Baseline demographic, clinical, echocardiographic, angiographic, patient-reported, and medication characteristics were compared between the CTO-PCI + OMT and OMT groups. Continuous variables were compared using the independent-samples Student’s *t*-test or the Mann–Whitney U test, according to distribution. Categorical variables were compared using Pearson’s χ^2^ test, Fisher’s exact test, or the Fisher–Freeman–Halton exact test, as appropriate. For comparisons involving more than two independent groups, the Kruskal–Wallis test was used for non-normally distributed continuous or ordinal variables. Standardized mean differences were additionally calculated as descriptive measures of baseline balance, with absolute values >0.10 interpreted as potentially meaningful imbalance.

The proportion of patients undergoing dobutamine stress echocardiography was reported overall and according to treatment strategy. Between-group comparison was performed using Fisher’s exact test. Because quantitative ischemic burden and viability extent were not uniformly available, these variables were not included in the primary outcome models. Performance of dobutamine stress echocardiography was included as a measured baseline variable in the propensity-score model.

Medication use at baseline and 12 months was summarized according to treatment strategy. Categorical medication variables were reported as *n*/*N* (%), and between-group comparisons were performed using Fisher’s exact test. At 12 months, medication analyses were restricted to patients with available follow-up medication data. To describe overall treatment intensity, the number of antianginal medication classes was calculated as the sum of beta-blocker, calcium-channel blocker, nitrate, trimetazidine, ranolazine, and ivabradine use. The number of secondary-prevention medication classes was calculated as the sum of aspirin, P2Y12 receptor inhibitor, angiotensin-converting enzyme inhibitor, angiotensin II receptor blocker, statin, and other lipid-lowering drug use. Medication-intensity counts were reported as mean ± SD and compared between groups descriptively.

Within-group changes from baseline to 12-month follow-up were assessed separately in each treatment group. For continuous variables measured at two time points, paired-samples *t*-tests or Wilcoxon signed-rank tests were used, depending on distribution. Changes in paired categorical data were analyzed using McNemar’s test when appropriate. Changes in Canadian Cardiovascular Society (CCS) angina class were analyzed as ordinal categorical data using non-parametric or paired categorical methods, as appropriate.

The primary patient-reported analysis was based on Seattle Angina Questionnaire (SAQ) domain scores assessed at baseline and at 12-month follow-up. The primary SAQ analyses were conducted in the complete-case SAQ population, defined as patients with available baseline and 12-month SAQ assessments for all five SAQ domains. The number and proportion of patients with missing 12-month SAQ data were reported overall and according to treatment strategy. Reasons for missing SAQ follow-up were categorized as death before 12-month SAQ reassessment, loss to SAQ follow-up, incomplete questionnaire, or administrative missingness.

To assess the potential impact of missing SAQ follow-up, baseline characteristics of patients with complete versus missing SAQ data were compared. Treatment-group differences in SAQ missingness were assessed using Fisher’s exact test. Continuous baseline characteristics were compared using independent-samples *t*-tests or Mann–Whitney U tests, as appropriate, and categorical variables were compared using χ^2^ or Fisher’s exact tests.

No multiple imputation of continuous 12-month SAQ domain scores was performed because missingness was mainly attributable to death before SAQ reassessment or loss to follow-up and therefore was not considered missing completely at random. In particular, imputing patient-reported health-status scores after death was not considered clinically meaningful. Instead, complete-case analyses were supplemented by missing-data characterization and by a conservative full-cohort responder sensitivity analysis for SAQ Angina Frequency. In this sensitivity analysis, patients without complete 12-month SAQ Angina Frequency data were classified as not having achieved clinically meaningful improvement.

For each SAQ domain, absolute change was calculated as the 12-month score minus the baseline score. Between-group differences in change were reported as mean differences with 95% confidence intervals. To address baseline imbalance, baseline-adjusted analysis of covariance models were fitted for each SAQ domain, with the 12-month SAQ domain score as the dependent variable, treatment strategy as the main independent variable, and the corresponding baseline SAQ domain score as a covariate.

Clinically meaningful improvement was assessed descriptively across all SAQ domains using three thresholds: ≥5-point, ≥10-point, and ≥20-point improvement from baseline to 12 months. For the principal symptom-specific domain, SAQ Angina Frequency, clinically meaningful improvement was additionally analyzed using logistic regression.

To account for baseline differences in SAQ Angina Frequency, an adjusted linear regression model was fitted with 12-month SAQ Angina Frequency as the dependent variable. Treatment strategy was entered as the main independent variable, with CTO-PCI + OMT compared with OMT alone. The model was adjusted for baseline SAQ Angina Frequency, age, sex, baseline left ventricular ejection fraction, history of smoking, previous PCI, and J-CTO score. Regression coefficients were reported as β estimates with 95% confidence intervals.

In an exploratory analysis, clinically meaningful improvement in SAQ Angina Frequency was defined as an increase of at least 10 points from baseline to 12 months. This binary outcome was analyzed using logistic regression. Odds ratios with 95% confidence intervals were reported. The baseline-adjusted logistic model included treatment strategy and baseline SAQ Angina Frequency. The extended model additionally included age, sex, baseline LVEF, history of smoking, previous PCI, and J-CTO score. Sensitivity analyses additionally included RCA target-vessel involvement and three-vessel coronary artery disease to assess whether angiographic imbalance influenced the association between treatment strategy and 12-month SAQ Angina Frequency.

To further address measured treatment-selection imbalance, a propensity-score weighting sensitivity analysis was performed. The propensity score was defined as the estimated probability of being managed with CTO-PCI + OMT rather than OMT alone. Propensity scores were estimated using a multivariable logistic regression model including baseline demographic, clinical, echocardiographic, angiographic, patient-reported, and treatment-intensity variables selected a priori on the basis of clinical relevance and reviewer-identified imbalance. These variables included age, sex, hypertension, dyslipidemia, diabetes mellitus, previous stroke, peripheral artery disease, chronic kidney disease, chronic obstructive pulmonary disease, atrial fibrillation, smoking history, previous myocardial infarction, previous PCI, previous CABG, baseline LVEF, baseline CCS class, baseline SAQ domains, RCA CTO, LAD CTO, J-CTO score, three-vessel coronary artery disease, dobutamine stress echocardiography, baseline antianginal medication count, and baseline secondary-prevention medication count. LCX CTO was assessed in the covariate-balance table but was not included in the final propensity-score model to avoid instability from simultaneous inclusion of multiple target-vessel indicators.

Conventional stabilized inverse probability of treatment weighting was evaluated but produced extreme weights and a substantially reduced effective sample size, indicating limited stability for the present dataset. Therefore, propensity-score overlap weighting was selected as the primary propensity-based sensitivity analysis. Overlap weights were defined as 1−propensity score for patients in the CTO-PCI + OMT group and as the propensity score for patients in the OMT group. Covariate balance before and after weighting was assessed using standardized mean differences, with absolute values < 0.10 considered acceptable.

The propensity-score overlap-weighted analyses repeated the main comparative models for 12-month SAQ Angina Frequency, change in SAQ Angina Frequency from baseline to 12 months, clinically meaningful improvement in SAQ Angina Frequency, cardiovascular rehospitalization, and exploratory LVEF assessment. Weighted linear regression was used for continuous outcomes, and weighted logistic regression was used for binary outcomes. Robust standard errors were used for weighted models. Results were reported as β estimates or odds ratios with 95% confidence intervals. These analyses were considered sensitivity analyses and were interpreted as adjusted associations rather than causal treatment effects.

For the treatment-strategy comparison, patients were analyzed according to the therapeutic strategy selected at baseline. Thus, patients in whom a CTO-PCI strategy was undertaken were analyzed in the CTO-PCI + OMT group regardless of final procedural success. Procedural success and periprocedural complications were analyzed separately among patients in the CTO-PCI + OMT group. For key procedural proportions, 95% confidence intervals were calculated using the Wilson method.

A secondary exploratory descriptive analysis compared three groups: patients with successful CTO-PCI, patients with failed CTO-PCI, and patients managed with OMT alone. Because procedural success is a post-treatment variable, this analysis was considered descriptive and hypothesis-generating only. It was not used for primary treatment-effect inference. No multivariable or propensity-weighted modeling was performed for this three-group comparison because the failed CTO-PCI subgroup was small and because procedural success may be influenced by lesion complexity, procedural course, operator judgment, and other post-baseline factors. Continuous variables were compared descriptively using the Kruskal–Wallis test, and categorical variables were compared using χ^2^ or Fisher–Freeman–Halton exact testing when appropriate.

Procedural and angiographic outcomes were summarized descriptively among patients in the CTO-PCI + OMT group. Categorical procedural variables were reported as *n*/*N* (%) with 95% confidence intervals calculated using the Wilson method. Because procedural metrics were available only for patients in whom CTO-PCI was attempted, these analyses were not used for between-group comparison with the OMT group. Detailed procedural variables that were not uniformly available were described as unavailable and included in the limitations.

Bleeding outcomes were not analyzed as formal comparative endpoints because bleeding events during DAPT were not systematically adjudicated according to BARC criteria in the available dataset. Therefore, no multivariable bleeding model was performed.

Exploratory adjusted linear regression was used to assess the association between treatment strategy and 12-month LVEF. The model included treatment strategy as the main independent variable and was adjusted for baseline LVEF, age, sex, smoking history, previous PCI, and J-CTO score. A sensitivity model additionally included RCA target-vessel involvement and three-vessel coronary artery disease. Propensity-score overlap-weighted exploratory analysis adjusted for baseline LVEF was also performed as a sensitivity analysis. Because LVEF was a secondary exploratory outcome and small echocardiographic differences may fall within expected measurement variability, interpretation emphasized the absolute magnitude and confidence interval of the effect estimate rather than statistical significance alone.

Cardiovascular rehospitalization was analyzed as a cumulative 12-month binary outcome. Between-group differences were assessed using χ^2^ or Fisher’s exact testing, as appropriate. Relative risk, unadjusted odds ratio, absolute risk difference, and number needed to treat were calculated for descriptive comparison between CTO-PCI + OMT and OMT alone. The estimated number needed to treat was interpreted descriptively and not as a causal treatment-effect estimate because treatment allocation was non-randomized.

Time-to-event analysis for cardiovascular rehospitalization was not performed because exact dates of rehospitalization were not consistently available for all patients. Therefore, Kaplan–Meier curves and Cox proportional hazards models were restricted to survival outcomes, for which time-to-event information and censoring times were available.

Exploratory logistic regression models were used to evaluate the association between treatment strategy and cardiovascular rehospitalization. The baseline-adjusted model included treatment strategy and baseline SAQ Quality of Life, whereas the extended model additionally included age, sex, baseline LVEF, smoking history, previous PCI, and J-CTO score. A propensity-score overlap-weighted logistic regression model was additionally performed as a sensitivity analysis, as described above. Because event numbers were modest, all rehospitalization models were interpreted as exploratory.

Time-to-event outcomes, including all-cause mortality, were evaluated using Kaplan–Meier survival analysis. Survival curves were compared using the log-rank test, with Breslow and Tarone–Ware tests used as complementary analyses when appropriate. Patients lost to follow-up were censored at the last available contact. Because the number of deaths was limited, survival analyses were considered exploratory and were not powered to detect realistic differences in mortality between treatment strategies. Multivariable Cox proportional hazards regression was not performed because the limited number of deaths would have created a high risk of model overfitting. Survival results were reported descriptively and were not interpreted as evidence of either prognostic benefit or absence of benefit.

Because this was a single-center, non-randomized retrospective observational cohort analysis, all comparative and multivariable analyses were interpreted as associations rather than proof of causality. Subgroup, sensitivity, secondary endpoint, and exploratory analyses were considered hypothesis-generating. No formal adjustment for multiple comparisons was applied. Therefore, *p*-values for secondary and exploratory analyses should be interpreted as nominal. Findings from these analyses were interpreted in the context of multiple comparisons and the potential for type I error. Accordingly, emphasis was placed on effect sizes, 95% confidence intervals, consistency across analyses, and clinical plausibility rather than isolated *p*-values.

## 3. Results

### 3.1. Study Population and Baseline Characteristics

**Figure 1:**
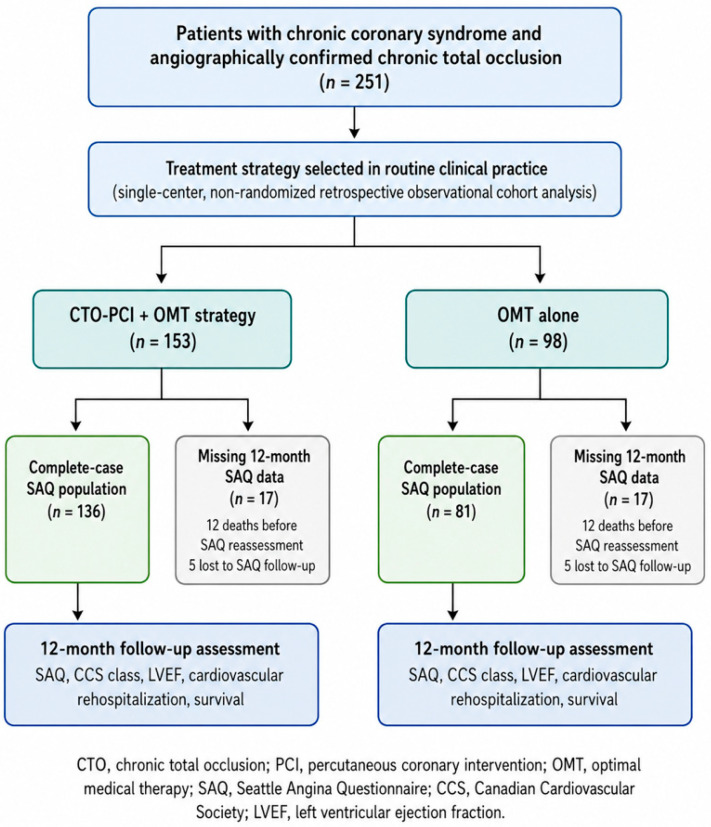
Study flow diagram. A total of 251 patients with chronic coronary syndrome and angiographically confirmed chronic total coronary occlusion were included in this single-center, non-randomized retrospective observational cohort analysis. Patients were categorized according to the treatment strategy selected in routine clinical practice: CTO-PCI + OMT or OMT alone. The complete-case SAQ population included 217 patients with available baseline and 12-month SAQ data. Thirty-four patients lacked complete 12-month SAQ data because of death before SAQ reassessment (*n* = 24) or loss to SAQ follow-up (*n* = 10).

A total of 251 patients with chronic coronary syndrome and angiographically confirmed chronic total coronary occlusion were included in this single-center, non-randomized retrospective observational cohort analysis. Of these, 153 patients (61.0%) were managed with an interventional treatment strategy consisting of attempted CTO-PCI in addition to optimal medical therapy (CTO-PCI + OMT), whereas 98 patients (39.0%) were managed with OMT alone. Patients in the OMT group were managed conservatively despite technically conceivable CTO-PCI, whereas patients in whom CTO anatomy was considered unsuitable for any reasonable attempt at safe and effective PCI were not included in the analytic cohort. The overall study flow, treatment-strategy groups, complete-case SAQ population, and reasons for missing 12-month SAQ follow-up are shown in Figure 1.

Baseline demographic and clinical characteristics according to treatment strategy are summarized in Table 1.

The two treatment-strategy groups were broadly comparable with respect to age and sex. Mean age was 66.26 ± 9.68 years in the CTO-PCI + OMT group and 68.55 ± 9.68 years in the OMT group, corresponding to a mean difference of −2.29 years (*p* = 0.069). Men represented 80.4% of the CTO-PCI + OMT group and 76.5% of the OMT group, corresponding to an absolute difference of +3.9 percentage points (*p* = 0.527).

The study population had a high burden of cardiovascular risk factors and comorbidities, consistent with advanced coronary artery disease. Hypertension was present in nearly all patients in both groups. Dyslipidemia, diabetes mellitus, peripheral artery disease, chronic kidney disease, chronic obstructive pulmonary disease, atrial fibrillation, and previous myocardial infarction were also common and were broadly distributed between treatment strategies.

Several clinically relevant baseline differences were observed. A history of smoking was more frequent in the OMT group than in the CTO-PCI + OMT group (52.0% vs. 34.6%; absolute difference, −17.4 percentage points for CTO-PCI + OMT vs. OMT; *p* = 0.008). In contrast, previous PCI was more frequent in the CTO-PCI + OMT group than in the OMT group (81.0% vs. 63.3%; absolute difference, +17.7 percentage points; *p* = 0.002). Previous myocardial infarction was common in both groups (75.2% vs. 64.3%; absolute difference, +10.9 percentage points; *p* = 0.087). Previous stroke and previous CABG were numerically more frequent in the OMT group, although these comparisons were interpreted descriptively.

These baseline differences highlight the non-randomized nature of treatment-strategy selection in routine clinical practice and support the need for adjustment for measured confounding. Therefore, clinically relevant baseline variables, including smoking history, previous PCI, baseline symptom status, baseline LVEF, angiographic characteristics, and CTO complexity, were considered in subsequent multivariable and propensity-score overlap-weighted analyses. Covariate balance before and after propensity-score overlap weighting is reported separately in Supplementary Table S1.

### 3.2. Angiographic Characteristics

Baseline angiographic characteristics according to treatment strategy are summarized in Table 2.

CTO target-vessel distribution differed between the treatment groups. Right coronary artery CTO was more frequent in the OMT group than in the CTO-PCI + OMT group (71/98 [72.4%] vs. 85/153 [55.6%]), corresponding to an absolute difference of −16.8 percentage points for CTO-PCI + OMT versus OMT (*p* = 0.008). Left anterior descending artery CTO was numerically more frequent in the CTO-PCI + OMT group (36/153 [23.5%] vs. 15/98 [15.3%]; absolute difference, +8.2 percentage points; *p* = 0.148). Left circumflex CTO was also numerically more frequent in the CTO-PCI + OMT group (33/153 [21.6%] vs. 12/98 [12.2%]; absolute difference, +9.4 percentage points; *p* = 0.065). No left main CTO was recorded in either group.

Lesion complexity assessed by J-CTO difficulty category was broadly comparable between treatment strategies. Difficult or very difficult CTO lesions, defined as J-CTO score ≥ 2, were present in 84 of 153 patients (54.9%) in the CTO-PCI + OMT group and 62 of 98 patients (63.3%) in the OMT group, corresponding to an absolute difference of −8.4 percentage points (*p* = 0.535 for the overall between-group comparison of J-CTO complexity).

The extent of multivessel coronary artery disease was also similar between groups. Three-vessel coronary artery disease was present in 100 of 153 patients (65.4%) in the CTO-PCI + OMT group and 70 of 98 patients (71.4%) in the OMT group, corresponding to an absolute difference of −6.0 percentage points (*p* = 0.336).

Overall, both treatment groups had a high burden of complex coronary artery disease, with broadly comparable CTO lesion complexity and extent of multivessel disease. The main angiographic imbalance was the higher frequency of RCA CTO in the OMT group. Target-vessel distribution, J-CTO complexity, and three-vessel coronary artery disease were therefore considered in subsequent multivariable, sensitivity, and propensity-score overlap-weighted analyses.

### 3.3. Ischemia and Viability Assessment

Evidence supporting viability in the CTO-related territory was required as part of routine clinical decision-making. Dobutamine stress echocardiography was the functional ischemia/viability testing modality systematically captured in the available dataset. Overall, dobutamine stress echocardiography was performed in 93 of 251 patients (37.1%).

According to treatment strategy, dobutamine stress echocardiography was performed in 51 of 153 patients (33.3%) in the CTO-PCI + OMT group and in 42 of 98 patients (42.9%) in the OMT group. This corresponded to an absolute difference of −9.5 percentage points for CTO-PCI + OMT versus OMT and was not statistically significant (*p* = 0.142). Documented ischemia and viability assessment according to treatment strategy is summarized in Supplementary Table S2.

Quantitative ischemic burden and quantitative viability extent were not uniformly available and therefore could not be compared between treatment groups. Similarly, other advanced functional imaging modalities, including stress cardiac magnetic resonance, nuclear perfusion imaging, and positron-emission tomography, were not systematically captured in the dataset. Therefore, the contribution of ischemic burden and viability extent to treatment selection and outcomes could not be fully evaluated.

### 3.4. Propensity-Score Balance Assessment

A propensity-score model was developed to address measured baseline imbalance between the non-randomized treatment-strategy groups. The model included baseline demographic, clinical, echocardiographic, angiographic, patient-reported, and treatment-intensity variables considered clinically relevant for treatment selection. The model showed good discrimination for treatment selection, with a c-statistic of 0.862.

Conventional stabilized inverse probability of treatment weighting was evaluated but produced extreme weights, with a maximum stabilized weight of 33.5 and an effective sample size of approximately 45 patients. This indicated limited stability for conventional IPTW in the present dataset. Therefore, propensity-score overlap weighting was selected as the primary propensity-based sensitivity approach.

Before weighting, several clinically relevant covariates showed meaningful imbalance between treatment groups, including baseline SAQ Angina Frequency, previous PCI, baseline SAQ Treatment Satisfaction, baseline secondary-prevention medication count, baseline SAQ Physical Limitation, RCA CTO, smoking history, baseline antianginal medication count, and baseline LVEF.

After propensity-score overlap weighting, covariate balance improved substantially. The maximum absolute standardized mean difference decreased from 0.451 before weighting to 0.033 after weighting, and no assessed covariate had an absolute standardized mean difference > 0.10 after weighting. The effective sample size after overlap weighting was 149.4 in the full cohort and 133.6 in the complete-case SAQ population. Baseline covariate balance before and after overlap weighting is shown in Supplementary Table S1.

These balance results supported the use of propensity-score overlap-weighted sensitivity analyses for the main patient-reported outcome, cardiovascular rehospitalization, and exploratory LVEF assessment. However, because propensity-score weighting can account only for measured variables, residual confounding by indication and unmeasured treatment-selection factors remain possible.

### 3.5. Procedural and Angiographic Outcomes and Periprocedural Safety

Procedural and angiographic outcomes in the CTO-PCI + OMT group are summarized in Table 3. Among the 153 patients in whom an interventional CTO-PCI strategy was undertaken, successful recanalization of the target CTO was achieved in 129 patients, corresponding to an overall technical success rate of 84.3% (95% CI, 77.7–89.2). CTO-PCI was unsuccessful in 24 patients (15.7%).

According to the recorded procedural fields, favorable post-procedural TIMI flow was documented in 146 of 153 patients (95.4%), and favorable residual stenosis after PCI was documented in 147 of 153 patients (96.1%). These angiographic component measures were interpreted descriptively and should be considered alongside the stricter technical success definition, which required successful recanalization of the target CTO with restoration of TIMI grade 3 antegrade flow and residual stenosis < 30% in the treated segment.

Periprocedural complications were infrequent but clinically relevant. At least one recorded periprocedural complication occurred in 13 of 153 patients (8.5%). Procedural myocardial infarction occurred in seven patients (4.6%), coronary perforation in five patients (3.3%), cardiac tamponade in two patients (1.3%), and stroke in one patient (0.7%). No periprocedural death and no need for urgent coronary artery bypass grafting were recorded.

Overall, the CTO-PCI strategy was associated with a high technical success rate and a low rate of serious recorded periprocedural complications in this selected cohort. However, detailed procedural metrics such as access site, antegrade versus retrograde crossing strategy, use of dissection/re-entry, contrast volume, fluoroscopy time, radiation dose, stent characteristics, and procedural duration were not uniformly available and therefore could not be analyzed. Procedural outcomes were analyzed only within the CTO-PCI + OMT group, as patients in the OMT group did not undergo attempted CTO-PCI at baseline.

### 3.6. Medical Therapy at Baseline and 12-Month Follow-Up

Medical therapy at baseline and 12-month follow-up is summarized in Table 4. Both treatment groups received active guideline-based medical therapy, including antiplatelet therapy, lipid-lowering therapy, antianginal therapy, and pharmacological treatment of cardiovascular risk factors.

At baseline, aspirin and P2Y12 receptor inhibitor use were more frequent in the CTO-PCI + OMT group, whereas nitrate use was more frequent in the OMT group. Aspirin was used in 147 of 153 patients (96.1%) in the CTO-PCI + OMT group and 84 of 98 patients (85.7%) in the OMT group (*p* = 0.004). P2Y12 receptor inhibitor use was also more frequent in the CTO-PCI + OMT group (150/153 [98.0%] vs. 77/98 [78.6%]; *p* < 0.001). In contrast, nitrate therapy was more frequent in the OMT group (46/98 [46.9%] vs. 45/153 [29.4%] in the CTO-PCI + OMT group; *p* = 0.007).

Baseline beta-blocker, angiotensin-converting enzyme inhibitor, angiotensin II receptor blocker, calcium-channel blocker, statin, other lipid-lowering drugs, trimetazidine, ranolazine, and ivabradine use were broadly comparable between groups. The mean number of antianginal medication classes was higher in the OMT group than in the CTO-PCI + OMT group (2.08 ± 1.00 vs. 1.75 ± 1.01; *p* = 0.012), whereas the mean number of secondary-prevention medication classes was higher in the CTO-PCI + OMT group (3.81 ± 0.62 vs. 3.51 ± 0.92; *p* = 0.005).

At 12 months, P2Y12 receptor inhibitor use remained more frequent in the CTO-PCI + OMT group (115/135 [85.2%] vs. 50/81 [61.7%]; *p* < 0.001), consistent with antiplatelet treatment after PCI and stent implantation. Nitrate use remained more frequent in the OMT group (43/81 [53.1%] vs. 39/135 [28.9%]; *p* < 0.001), reflecting ongoing symptom-directed antianginal management in conservatively treated patients.

Statin use remained high in both groups at 12 months, and use of additional lipid-lowering therapy increased modestly during follow-up. The mean number of antianginal medication classes remained higher in the OMT group (2.33 ± 1.19 vs. 1.89 ± 1.12; *p* = 0.007). The mean number of secondary-prevention medication classes was numerically higher in the CTO-PCI + OMT group at 12 months, but the difference was smaller than at baseline (3.51 ± 0.79 vs. 3.30 ± 0.86; *p* = 0.069).

Overall, these findings indicate that OMT alone represented an active medical treatment strategy rather than absence of therapy. However, medication patterns were not identical between groups. Because medication dose, adherence, reasons for treatment modification, LDL-C control, blood-pressure control, and glycemic control were not uniformly available, residual differences in medical optimization cannot be excluded.

### 3.7. Completeness of SAQ Follow-Up and Missing-Data Assessment

Of the 251 patients included in the study, 217 patients had complete baseline and 12-month SAQ data for all five SAQ domains and were included in the complete-case SAQ population. This included 136 of 153 patients in the CTO-PCI + OMT group and 81 of 98 patients in the OMT group. Thirty-four patients lacked complete 12-month SAQ data, corresponding to 13.5% of the overall cohort.

The reasons for missing 12-month SAQ data were death before SAQ reassessment in 24 patients and loss to SAQ follow-up in 10 patients. No missing SAQ follow-up was attributable to incomplete questionnaires or administrative missingness. Missing SAQ follow-up occurred in 17 patients in the CTO-PCI + OMT group and 17 patients in the OMT group, corresponding to 11.1% and 17.3% of the respective treatment groups (*p* = 0.187). In each treatment group, 12 patients died before 12-month SAQ reassessment, and 5 patients were lost to SAQ follow-up.

Patients with missing SAQ follow-up differed from patients with complete SAQ data in several clinically relevant baseline characteristics. They were older, had lower baseline LVEF, and had a higher prevalence of atrial fibrillation. Baseline SAQ Angina Frequency was similar between patients with complete and missing SAQ follow-up. Baseline characteristics according to SAQ follow-up completeness are shown in Supplementary Table S3.

The survival analysis reported 11 patients lost to follow-up because one patient had complete 12-month SAQ data but incomplete vital-status follow-up and was therefore censored in the survival analysis. Thus, the number of patients lost specifically to SAQ follow-up was 10, whereas the number lost for survival follow-up was 11.

Because missing SAQ follow-up was mainly attributable to death or loss to follow-up, missingness was not considered completely random. Therefore, complete-case SAQ analyses were interpreted in the context of potential survivorship bias. A conservative full-cohort responder sensitivity analysis for SAQ Angina Frequency, in which patients without complete 12-month SAQ data were classified as not having achieved clinically meaningful improvement, is reported below with the SAQ Angina Frequency analyses.

### 3.8. Patient-Reported Health Status Assessed by the Seattle Angina Questionnaire

The primary patient-reported analysis was performed in the complete-case SAQ population, including 217 patients with complete baseline and 12-month data for all five SAQ domains: 136 patients in the CTO-PCI + OMT group and 81 patients in the OMT group. The overall SAQ assessment was considered the primary patient-reported framework, while SAQ Angina Frequency was the principal symptom-specific domain for adjusted comparative analysis. Changes in all five SAQ domains are summarized in Table 5.

Both treatment strategies were associated with improvement across all SAQ domains from baseline to 12 months. However, baseline differences were present in several domains, particularly Physical Limitation, Angina Frequency, and Treatment Satisfaction. Therefore, absolute follow-up comparisons were interpreted descriptively, and the interpretation emphasized change-score analyses and baseline-adjusted models.

In the Physical Limitation domain, the CTO-PCI + OMT group had higher absolute scores at both baseline and follow-up. However, the mean change from baseline was not greater with CTO-PCI + OMT than with OMT alone (+4.98 ± 11.03 vs. +6.35 ± 12.48 points; between-group Δ difference, −1.37 points; 95% CI, −4.69 to 1.94; *p* = 0.415). Therefore, the unadjusted 12-month Physical Limitation difference should be interpreted cautiously and should not be considered evidence of greater functional improvement with CTO-PCI + OMT.

SAQ Angina Frequency improved substantially in both groups. The mean improvement was +21.54 ± 18.37 points in the CTO-PCI + OMT group and +20.00 ± 19.36 points in the OMT group. The unadjusted between-group difference in change was small and not statistically significant (+1.54 points; 95% CI, −3.72 to 6.81; *p* = 0.563). However, because baseline Angina Frequency was lower in the OMT group, baseline-adjusted and propensity-score overlap-weighted models were used for the principal symptom-specific comparative analysis.

Angina Stability improved in both groups, with a mean change of +14.71 ± 30.35 points in the CTO-PCI + OMT group and +12.04 ± 27.42 points in the OMT group. The between-group difference in change was +2.67 points (95% CI, −5.24 to 10.57; *p* = 0.506).

Treatment Satisfaction also improved in both treatment groups. The mean change was +6.87 ± 15.91 points in the CTO-PCI + OMT group and +11.11 ± 14.35 points in the OMT group, corresponding to a between-group difference in change of −4.24 points (95% CI, −8.38 to −0.10; *p* = 0.045). Because multiple SAQ domains were evaluated and baseline differences were present, this domain-level finding was interpreted descriptively.

Quality of Life improved similarly in both groups. The mean change was +11.85 ± 18.83 points in the CTO-PCI + OMT group and +12.55 ± 17.03 points in the OMT group, corresponding to a between-group difference in change of −0.71 points (95% CI, −5.61 to 4.20; *p* = 0.777).

In baseline-adjusted domain-level models, SAQ Angina Frequency was the only SAQ domain with a statistically significant 12-month between-group difference favoring CTO-PCI + OMT (β = 7.27 points; 95% CI, 3.72–10.82; *p* < 0.001). In contrast, baseline-adjusted differences were not significant for Physical Limitation, Angina Stability, Treatment Satisfaction, or Quality of Life. These findings support the analytic focus on SAQ Angina Frequency as the principal symptom-specific SAQ domain. Baseline-adjusted domain-level models are presented in Supplementary Table S4.

Clinically meaningful improvement analyses are shown in Supplementary Table S5. In the principal symptom-specific domain, SAQ Angina Frequency, ≥10-point improvement occurred in 116 of 136 patients (85.3%) in the CTO-PCI + OMT group and 59 of 81 patients (72.8%) in the OMT group. Large improvement, defined as ≥20 points, occurred in 92 of 136 patients (67.6%) and 46 of 81 patients (56.8%), respectively.

Overall, both treatment strategies were associated with broad improvement in patient-reported health status during follow-up. The domain-level change-score analysis showed substantial improvement in both groups, while the principal adjusted between-group signal favoring CTO-PCI + OMT was observed in SAQ Angina Frequency.

### 3.9. Adjusted and Propensity-Score Overlap-Weighted Analysis of SAQ Angina Frequency

SAQ Angina Frequency was analyzed as the principal symptom-specific SAQ domain because it directly captures anginal symptom frequency and the need for symptom-relieving medication. This domain also showed the clearest baseline-adjusted between-group difference among the SAQ domains, whereas other domains were interpreted as secondary and exploratory. The results of the multivariable, propensity-score overlap-weighted, and conservative responder sensitivity analyses are presented in Table 6.

In the multivariable ANCOVA model, CTO-PCI + OMT was associated with a 7.07-point higher 12-month SAQ Angina Frequency score compared with OMT alone after adjustment for baseline SAQ Angina Frequency and relevant clinical and angiographic covariates (β = 7.07; 95% CI, 3.36–10.79; *p* < 0.001). Baseline SAQ Angina Frequency was also associated with the 12-month Angina Frequency score (β = 0.29 per point; 95% CI, 0.18–0.40; *p* < 0.001). J-CTO score was associated with a slightly higher 12-month Angina Frequency score (β = 2.19 per point; 95% CI, 0.58–3.81; *p* = 0.008), whereas age, sex, baseline LVEF, smoking history, and previous PCI were not materially associated with the outcome in this model.

In the propensity-score overlap-weighted sensitivity analysis, baseline SAQ Angina Frequency was well balanced between the two treatment groups. The weighted mean baseline SAQ Angina Frequency was 68.12 in the CTO-PCI + OMT group and 67.87 in the OMT group. At 12 months, the corresponding weighted mean values were 90.77 and 84.14, respectively.

In the propensity-score overlap-weighted ANCOVA model adjusted for baseline SAQ Angina Frequency, CTO-PCI + OMT remained associated with higher 12-month SAQ Angina Frequency compared with OMT alone (β = 6.55 points; 95% CI, 2.39–10.70; *p* = 0.002). A propensity-score overlap-weighted change-score analysis yielded a directionally consistent between-group difference of 6.39 points in favor of CTO-PCI + OMT (95% CI, 0.53–12.24; *p* = 0.033). The weighted mean improvement in SAQ Angina Frequency was 22.65 points in the CTO-PCI + OMT group and 16.26 points in the OMT group.

Clinically meaningful improvement in SAQ Angina Frequency, defined as an increase of at least 10 points from baseline to 12 months, was more frequent with CTO-PCI + OMT in adjusted analyses. In the baseline-adjusted logistic regression model, CTO-PCI + OMT was associated with higher odds of achieving ≥10-point SAQ Angina Frequency improvement compared with OMT alone (OR = 6.03; 95% CI, 2.34–15.48; *p* < 0.001). Higher baseline SAQ Angina Frequency was associated with lower odds of additional ≥10-point improvement (OR = 0.90 per point; 95% CI, 0.87–0.93; *p* < 0.001).

In the extended model adjusted for baseline SAQ Angina Frequency, age, sex, baseline LVEF, smoking history, previous PCI, and J-CTO score, CTO-PCI + OMT remained associated with higher odds of ≥10-point SAQ Angina Frequency improvement (OR = 6.46; 95% CI, 2.34–17.87; *p* < 0.001). In the propensity-score overlap-weighted logistic regression model adjusted for baseline SAQ Angina Frequency, the estimate was directionally consistent (OR = 6.61; 95% CI, 2.48–17.62; *p* < 0.001).

To assess the robustness of this finding to missing SAQ follow-up data, a conservative full-cohort responder sensitivity analysis was performed. In this analysis, all patients without complete 12-month SAQ Angina Frequency data were classified as not having achieved clinically meaningful improvement. Using the full cohort as the denominator, ≥10-point improvement in SAQ Angina Frequency occurred in 116 of 153 patients (75.8%) in the CTO-PCI + OMT group and 59 of 98 patients (60.2%) in the OMT group. This corresponded to an unadjusted OR of 2.07 (95% CI, 1.20–3.59; *p* = 0.011). In an extended logistic regression model adjusted for baseline SAQ Angina Frequency, age, sex, baseline LVEF, smoking history, previous PCI, and J-CTO score, CTO-PCI + OMT remained associated with higher odds of achieving ≥10-point SAQ Angina Frequency improvement (OR = 3.70; 95% CI, 1.79–7.63; *p* < 0.001).

The adjusted between-group difference in 12-month SAQ Angina Frequency was approximately 6.5–7 points across the multivariable and propensity-score overlap-weighted models. This exceeds the commonly used approximate 5-point threshold for small clinically meaningful change and approaches the range generally considered moderate improvement. However, because the study was open-label, non-randomized, and not sham-controlled, the magnitude of patient-reported benefit should be interpreted cautiously and may reflect both treatment-associated anti-anginal benefit and non-specific expectation effects.

Overall, the adjusted and propensity-score overlap-weighted analyses supported the principal patient-reported finding: CTO-PCI + OMT was associated with higher 12-month SAQ Angina Frequency and higher odds of clinically meaningful improvement. Because treatment allocation was non-randomized, these estimates should be interpreted as adjusted associations rather than causal treatment effects.

### 3.10. Angina Status According to CCS Class

Angina status according to the Canadian Cardiovascular Society (CCS) classification and secondary clinical outcomes are summarized in Table 7. Because several secondary outcomes were evaluated, *p*-values in this table should be interpreted as nominal and exploratory.

At baseline, the distribution of CCS angina class was broadly similar between the two treatment groups. Most patients had advanced angina symptoms at the index assessment, with CCS class III or IV present in 68.0% of patients in the CTO-PCI + OMT group and 70.4% of patients in the OMT group. No clinically meaningful baseline difference in CCS class distribution was observed between treatment strategies.

During follow-up, both groups demonstrated a substantial shift toward lower CCS classes. In the CTO-PCI + OMT group, the mean ordinal CCS score decreased from 2.83 at baseline to 0.76 at 12 months, whereas in the OMT group it decreased from 2.90 to 0.90. Within-group improvement was observed in both treatment groups.

Improvement by at least one CCS class occurred in 115 of 136 patients (84.6%) in the CTO-PCI + OMT group and 72 of 81 patients (88.9%) in the OMT group, corresponding to an absolute difference of −4.3 percentage points. Improvement by at least two CCS classes occurred in 96 of 136 patients (70.6%) and 56 of 81 patients (69.1%), respectively, corresponding to an absolute difference of +1.5 percentage points. These differences were small and did not suggest a clear between-group advantage in physician-assessed CCS improvement.

At 12 months, the proportion of patients with no angina symptoms, corresponding to CCS class 0, was numerically higher in the CTO-PCI + OMT group than in the OMT group (84/136 [61.8%] vs. 39/81 [48.1%]; absolute difference, +13.7 percentage points; *p* = 0.065). CCS class I was more frequent in the OMT group (17/136 [12.5%] vs. 19/81 [23.5%]; absolute difference, −11.0 percentage points for CTO-PCI + OMT vs. OMT; *p* = 0.040). No relevant between-group differences were observed for CCS class II, III, or IV.

Overall, CCS class improved substantially in both groups. However, the degree of CCS improvement was similar between treatment strategies, and category-level differences at 12 months should be interpreted descriptively. In contrast, SAQ Angina Frequency provided a more sensitive patient-reported measure for identifying adjusted differences in angina-related health status between treatment strategies.

### 3.11. Left Ventricular Ejection Fraction

Left ventricular ejection fraction (LVEF) at baseline and 12-month follow-up is summarized in Table 7. LVEF was analyzed as a secondary exploratory outcome. No separate figure was included for this subsection because the LVEF results are presented together with the other secondary clinical outcomes.

At baseline, mean LVEF was higher in the CTO-PCI + OMT group than in the OMT group (49.20 ± 10.52% vs. 46.00 ± 11.85%), corresponding to a mean between-group difference of +3.20 percentage points (*p* = 0.030). At 12-month follow-up, mean LVEF remained higher in the CTO-PCI + OMT group (50.19 ± 8.84% vs. 46.77 ± 9.63%), corresponding to a mean between-group difference of +3.42 percentage points (*p* = 0.006).

In the subgroup with available paired LVEF measurements at both baseline and 12 months, the CTO-PCI + OMT group showed a small mean increase in LVEF of +0.26 ± 3.03 percentage points, whereas the OMT group showed a mean decrease of −0.88 ± 3.68 percentage points. This corresponded to an unadjusted between-group difference in LVEF trajectory of approximately +1.14 percentage points (*p* = 0.020).

In an exploratory adjusted linear regression model including baseline LVEF, age, sex, smoking history, previous PCI, and J-CTO score, CTO-PCI + OMT was associated with a slightly higher 12-month LVEF value compared with OMT alone (β = 1.33 percentage points; 95% CI, 0.46–2.20; *p* = 0.003). In a sensitivity model additionally adjusted for RCA target-vessel involvement and three-vessel coronary artery disease, the estimate remained small (β = 1.14 percentage points; 95% CI, 0.27–2.02; *p* = 0.011). In the propensity-score overlap-weighted exploratory analysis adjusted for baseline LVEF, CTO-PCI + OMT was associated with a 1.23-percentage-point higher 12-month LVEF value (95% CI, 0.18–2.27; *p* = 0.021).

Overall, CTO-PCI + OMT was associated with a small exploratory difference in LVEF trajectory during follow-up. The absolute magnitude of the adjusted estimates was modest, approximately 1.1–1.3 percentage points, and should be interpreted cautiously because baseline LVEF differed between groups and small echocardiographic LVEF differences may fall within expected measurement variability. These findings should not be interpreted as definitive evidence of preserved or improved global systolic function.

### 3.12. Cardiovascular Rehospitalization

**Figure 2:**
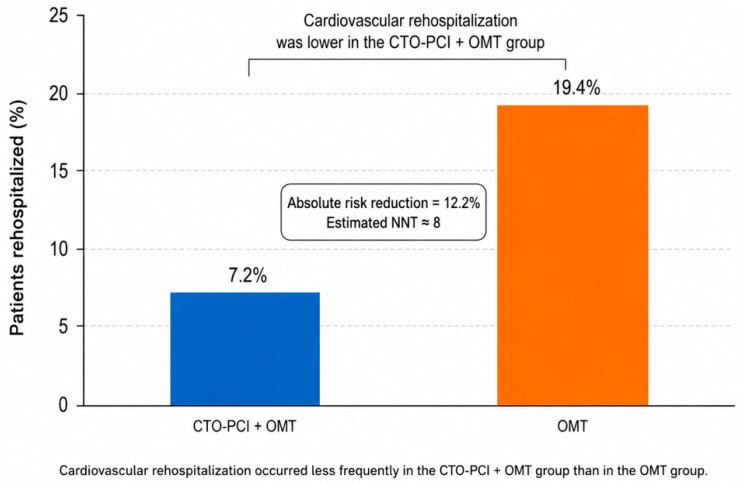
Cardiovascular rehospitalization during 12-month follow-up. Cardiovascular rehospitalization occurred less frequently in the CTO-PCI + OMT group than in the OMT group. The absolute risk reduction was 12.2%, corresponding to an estimated number needed to treat of approximately 8.

Cardiovascular rehospitalization was analyzed as a cumulative 12-month binary outcome and is summarized in Table 7 and Figure 2. Exact dates of cardiovascular rehospitalization were not consistently available for all patients; therefore, time-to-event analyses for this endpoint were not performed. Cardiovascular rehospitalization occurred in 11 of 153 patients (7.2%) in the CTO-PCI + OMT group and in 19 of 98 patients (19.4%) in the OMT group. This corresponded to an absolute risk difference of 12.2 percentage points, a relative risk of 0.37 (95% CI, 0.18–0.75), and an unadjusted odds ratio of 0.32 (95% CI, 0.15–0.71; *p* = 0.005).

The descriptive number needed to treat was approximately 8 over 12 months. However, this estimate should be interpreted descriptively rather than as a causal treatment-effect measure because treatment allocation was non-randomized, and rehospitalization was analyzed as a cumulative binary endpoint rather than a time-to-event endpoint.

In an exploratory logistic regression model adjusted for baseline SAQ Quality of Life, CTO-PCI + OMT was associated with lower odds of cardiovascular rehospitalization compared with OMT alone (OR = 0.31; 95% CI, 0.14–0.69; *p* = 0.004). Higher baseline SAQ Quality of Life was also associated with lower odds of rehospitalization (OR = 0.98 per point; 95% CI, 0.97–1.00; *p* = 0.024). In the extended exploratory model, additionally adjusted for age, sex, baseline LVEF, smoking history, previous PCI, and J-CTO score, the estimate remained similar (OR = 0.31; 95% CI, 0.13–0.76; *p* = 0.011).

In the propensity-score overlap-weighted sensitivity analysis, CTO-PCI + OMT was also associated with lower odds of cumulative 12-month cardiovascular rehospitalization (OR = 0.33; 95% CI, 0.13–0.86; *p* = 0.023). This sensitivity analysis supported the direction of the association after improving measured baseline balance. Rehospitalization models are summarized in Supplementary Table S6.

Because rehospitalization is partly symptom-driven and may be influenced by patient expectations, physician behavior, follow-up intensity, and thresholds for hospital admission, this outcome should be interpreted cautiously in the open-label observational setting. Overall, CTO-PCI + OMT was associated with lower cumulative 12-month cardiovascular rehospitalization, but this result should be interpreted as an adjusted association rather than definitive evidence of causal risk reduction.

### 3.13. Survival Outcomes

**Figure 3:**
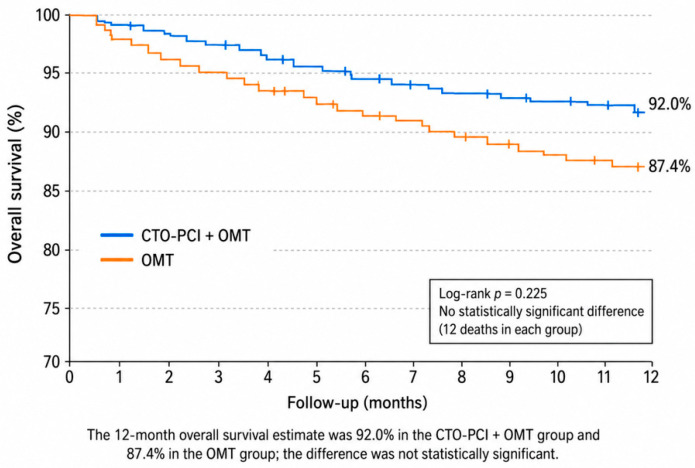
Kaplan–Meier analysis of overall survival according to treatment strategy. The 12-month overall survival estimate was 92.0% in the CTO-PCI + OMT group and 87.4% in the OMT group. The difference did not reach statistical significance by log-rank testing (*p* = 0.225).

Survival outcomes during 12-month follow-up are summarized in Table 7 and illustrated using Kaplan–Meier analysis in Figure 3. During follow-up, survival remained high in the overall cohort. The mean duration of follow-up was 11.37 ± 1.99 months. Eleven patients (4.4%) were lost to follow-up and were censored at the last available contact.

A total of 24 deaths were recorded, corresponding to 9.6% of the study population. Of these, 14 deaths were classified as cardiovascular and 10 deaths as non-cardiovascular. The 12-month overall survival rate in the total cohort was 90.4%, and the 12-month cardiovascular survival rate was 94.2%.

Kaplan–Meier analysis comparing the two treatment strategies showed a 12-month overall survival estimate of 92.0% in the CTO-PCI + OMT group and 87.4% in the OMT group. Twelve deaths occurred in each treatment group during follow-up. The log-rank test was not statistically significant (*p* = 0.225).

Because only 24 deaths were recorded in the overall cohort, the study was substantially underpowered to detect realistic differences in mortality between treatment strategies. Therefore, survival analyses should be interpreted as exploratory and should not be considered evidence of either prognostic benefit or absence of benefit. The main clinical interpretation of the study remains focused on patient-reported health status, SAQ Angina Frequency, and secondary clinical outcomes rather than mortality.

## 4. Discussion

### 4.1. Principal Findings

The present single-center, non-randomized retrospective observational cohort analysis evaluated patient-reported health status and 12-month clinical outcomes in patients with chronic coronary syndrome and angiographically confirmed chronic total occlusion managed with either CTO-PCI + OMT or OMT alone. The principal finding is that CTO-PCI + OMT was associated with greater improvement in disease-specific health status, particularly SAQ Angina Frequency. This association remained consistent after multivariable adjustment for baseline symptom status and relevant clinical and angiographic covariates, and was further supported by propensity-score overlap-weighted sensitivity analysis designed to improve measured baseline balance between the non-randomized treatment groups.

Both treatment strategies were associated with substantial improvement in SAQ domains and CCS angina class, emphasizing that OMT was an active therapeutic strategy rather than absence of treatment. Cardiovascular rehospitalization was observed less frequently in the CTO-PCI + OMT group, although this secondary endpoint should be interpreted cautiously because rehospitalization was analyzed as a cumulative binary outcome and may be influenced by treatment expectation, physician behavior, follow-up intensity, and access-to-care patterns. A small exploratory difference in LVEF trajectory was observed, but its magnitude was modest. The study was underpowered to evaluate mortality, and survival analyses should not be interpreted as evidence of either prognostic benefit or absence of benefit.

### 4.2. Interpretation of Patient-Reported Outcomes

The overall SAQ assessment was used as the primary patient-reported framework for evaluating disease-specific health status. Among the SAQ domains, Angina Frequency was considered the principal symptom-specific domain because it directly reflects anginal symptom frequency and use of symptom-relieving medication. This distinction is important because the expected benefit of CTO-PCI in chronic coronary syndrome is primarily symptomatic rather than prognostic over short-term follow-up.

Both CTO-PCI + OMT and OMT alone were associated with improvement across SAQ domains, which highlights the value of active medical therapy, symptom monitoring, and structured follow-up. However, baseline differences were present in several SAQ domains. Therefore, the revised analysis emphasizes change scores and baseline-adjusted models rather than relying only on absolute 12-month values. This is particularly relevant for Physical Limitation: although absolute follow-up scores were higher in the CTO-PCI + OMT group, change-score and baseline-adjusted analyses did not show a clear greater improvement with CTO-PCI + OMT. Physical Limitation should therefore be interpreted as a secondary descriptive finding.

In contrast, SAQ Angina Frequency remained the clearest symptom-specific signal favoring CTO-PCI + OMT. The adjusted between-group difference of approximately 6.5–7 points exceeded the approximate threshold for small clinically meaningful change and approached the range considered moderate improvement, while the within-group improvement after CTO-PCI exceeded thresholds commonly interpreted as large improvement. Nevertheless, because the study was open-label, non-randomized, and not sham-controlled, the magnitude of patient-reported benefit may reflect both treatment-associated anti-anginal effects and non-specific expectation effects.

### 4.3. Comparison with Randomized and Sham-Controlled Trials

The present findings are consistent with contemporary CTO evidence suggesting that the most reproducible benefit of CTO-PCI is improvement in symptoms and disease-specific health status, whereas prognostic benefit remains uncertain. EUROCTO demonstrated improvement in SAQ Angina Frequency and quality-of-life scores with CTO-PCI + OMT compared with OMT alone, while DECISION-CTO did not show superiority of routine CTO-PCI for major adverse cardiovascular outcomes [5,6]. These differences likely reflect patient selection, crossover, treatment of non-CTO lesions, baseline symptom burden, completeness of revascularization, and statistical power.

IMPACTOR-CTO and recent pooled and meta-analytic evidence further support the concept that CTO-PCI may improve ischemia-related and patient-centered outcomes in selected patients, while conclusions regarding death, myocardial infarction, and long-term prognosis remain more cautious [7,8,9,10]. The present analysis aligns with this framework: it supports an association with improved angina-related health status but does not establish or exclude a mortality benefit.

The sham-controlled ORBITA-CTO trial is particularly relevant because it addresses the placebo-sensitive nature of symptom endpoints [11]. Sham-controlled evidence supports a treatment-associated anti-anginal effect of CTO-PCI beyond placebo in selected patients. However, the magnitude of benefit observed in unblinded studies may exceed that observed under blinded conditions. Therefore, the approximately 6.5–7-point adjusted difference in SAQ Angina Frequency in the present study should be interpreted as an open-label adjusted patient-reported association, not as a sham-controlled efficacy estimate.

### 4.4. Rehospitalization, LVEF and Mortality: Cautious Interpretation

Cardiovascular rehospitalization was lower in the CTO-PCI + OMT group during 12-month follow-up. This endpoint is clinically relevant because it reflects recurrent symptoms, clinical deterioration, and healthcare resource use. However, exact dates of rehospitalization were not consistently available; therefore, this outcome was analyzed as a cumulative 12-month binary endpoint rather than as a time-to-event endpoint. The estimated number needed to treat should be interpreted descriptively rather than causally, because treatment allocation was non-randomized and rehospitalization may be affected by symptom perception, physician decision-making, follow-up intensity, and access-to-care patterns.

The LVEF findings also require caution. Although adjusted and propensity-score overlap-weighted exploratory analyses showed a small difference in 12-month LVEF trajectory favoring CTO-PCI + OMT, the absolute magnitude was approximately 1.1–1.3 percentage points. This is unlikely to represent a robust clinically meaningful improvement in global systolic function and may fall within expected echocardiographic measurement variability. Baseline LVEF differed between groups, and systematic blinding of echocardiographic readers to treatment strategy was not documented. Therefore, the LVEF results should be interpreted as secondary exploratory findings, not as definitive evidence of preserved or improved systolic function.

Survival analyses were substantially underpowered. Only 24 deaths occurred during follow-up, with 12 deaths in each treatment group. Accordingly, the absence of statistical significance should not be interpreted as evidence of no mortality difference. The present cohort and 12-month follow-up are insufficient for reliable mortality inference; the main contribution of the study lies in patient-reported and symptom-related outcomes.

### 4.5. Procedural Context and Safety

In the CTO-PCI + OMT group, technical success was achieved in 84.3% of patients, and serious recorded periprocedural complications were infrequent. These findings provide procedural context for interpreting the patient-reported results, because the potential value of CTO-PCI depends on whether symptom benefit can be achieved with acceptable procedural risk. However, CTO-PCI remains a technically complex intervention, and outcomes are closely linked to patient selection, lesion complexity, operator experience, and institutional expertise.

The available dataset included technical success, post-procedural TIMI flow, residual stenosis after PCI, and selected in-hospital complications. However, more granular procedural metrics—including access site, antegrade versus retrograde strategy, use of dissection/re-entry, contrast volume, fluoroscopy time, radiation dose, stent characteristics, and procedural duration—were not uniformly available. This limits assessment of procedural complexity, radiation and contrast burden, and strategy-specific safety.

The primary analysis remained treatment-strategy based, with all patients selected for CTO-PCI analyzed in the CTO-PCI + OMT group regardless of procedural success. A secondary exploratory comparison of successful CTO-PCI, failed CTO-PCI, and OMT alone may provide useful procedural context, but it should be interpreted descriptively only. Because procedural success is a post-treatment variable, this comparison is vulnerable to post-treatment bias and should not be used to infer the causal effect of successful recanalization. In addition, bleeding events during DAPT were not systematically adjudicated according to BARC criteria, limiting formal assessment of net clinical benefit.

### 4.6. Clinical Implications

These findings support a selective and patient-centered approach to CTO management. CTO-PCI should not be viewed as a universal strategy for all patients with CTO. Rather, it should be considered selectively in symptomatic patients with demonstrable potential for symptomatic benefit, after integrating symptom burden, ischemia and viability assessment, coronary anatomy, lesion complexity, expected procedural success, comorbidities, bleeding risk, patient preference, and local operator expertise.

The revised SAQ analyses suggest that SAQ Angina Frequency may be particularly useful for identifying symptom-related benefit after CTO-PCI, whereas broader domains such as Physical Limitation, Treatment Satisfaction, and Quality of Life may be more strongly influenced by baseline status, comorbidity, medical optimization, and expectation effects. Structured SAQ assessment may therefore help quantify baseline symptom burden, support shared decision-making, and monitor response to either CTO-PCI + OMT or OMT alone.

OMT remains an active and appropriate treatment strategy when symptoms are limited, expected symptomatic benefit is uncertain, procedural complexity or risk is high relative to expected benefit, comorbidity burden is substantial, or the patient prefers conservative management. Patients without objective evidence of ischemia or viable myocardium in the CTO-related territory were not included in the present analytic cohort. Therefore, the key clinical question is not simply whether a CTO can be recanalized, but whether recanalization is likely to provide meaningful benefit for the individual patient.

### 4.7. Strengths and Limitations

The main strengths of this study include the clinically relevant real-world CTO cohort, direct comparison of two routine treatment strategies, structured SAQ assessment across multiple domains, and integration of patient-reported outcomes with procedural, echocardiographic, rehospitalization, and survival data. The revised analysis also adds baseline-adjusted SAQ domain models, change-score analyses, clinically meaningful improvement thresholds, propensity-score overlap weighting, standardized mean-difference balance assessment, missing-data characterization, medication reporting, and sensitivity analyses.

Several limitations should be acknowledged. First, this was a single-center, non-randomized retrospective observational cohort analysis, and treatment allocation was based on clinical, angiographic, functional, operator-related, and patient-related considerations rather than randomization. Confounding by indication, healthier patient selection, physician selection bias, and residual unmeasured confounding remain possible despite multivariable adjustment and propensity-score overlap weighting. Propensity-score methods can account only for measured variables and cannot address unmeasured factors such as frailty, ischemic burden, viability extent, lesion morphology, distal vessel quality, collateral suitability, operator judgment, medication adherence, and patient preference.

Second, SAQ analyses were primarily complete-case. Missing SAQ follow-up occurred in 13.5% of the cohort and was mainly attributable to death before 12-month SAQ reassessment or loss to SAQ follow-up. Patients with missing SAQ follow-up were older and had lower baseline LVEF, indicating that missingness was not completely random and that survivorship bias cannot be excluded. Conservative responder sensitivity analysis supported the direction of the main SAQ Angina Frequency finding, but complete-case analyses may still overestimate patient-reported improvement.

Third, the study was open-label and lacked a sham control. Patient expectations, treatment perception, physician perception, and follow-up behavior may have influenced symptom-driven outcomes, including SAQ Angina Frequency, CCS class, quality-of-life measures, and cardiovascular rehospitalization. Thus, the observed patient-reported benefit should be interpreted as an open-label association rather than a sham-controlled efficacy estimate.

These limitations mean that the findings should be considered hypothesis-generating and applicable primarily to selected patients with chronic coronary syndrome and CTO treated in centers with CTO-PCI expertise. Further multicenter studies with longer follow-up, standardized patient-reported outcome collection, systematic ischemia/viability and bleeding assessment, detailed procedural reporting, and stronger control for treatment-selection bias are needed.

## 5. Conclusions

In this single-center, non-randomized retrospective observational cohort analysis of patients with chronic coronary syndrome and angiographically confirmed chronic total occlusion, a CTO-PCI + OMT strategy was associated with greater improvement in disease-specific health status, particularly SAQ Angina Frequency, compared with OMT alone. This association remained consistent after baseline adjustment, multivariable adjustment, and propensity-score overlap-weighted sensitivity analysis, although residual confounding by indication remains an important limitation.

CTO-PCI + OMT was also associated with lower cumulative 12-month cardiovascular rehospitalization; however, this secondary finding should be interpreted cautiously because of the non-randomized design, modest event numbers, lack of time-to-event rehospitalization data, and potential expectation-, physician-, and care-related biases. The study was underpowered to evaluate mortality, and survival analyses should not be interpreted as evidence of either prognostic benefit or absence of benefit. A small exploratory difference in LVEF trajectory was observed, but its magnitude was modest and should not be interpreted as definitive evidence of preserved systolic function.

These findings support consideration of CTO-PCI as a patient-centered therapeutic option in carefully selected symptomatic patients with demonstrable potential for symptomatic benefit, rather than as a universal strategy for all patients with CTO. The comparison should be interpreted in the context of active medical therapy in both groups, complete-case SAQ analysis, open-label outcome assessment, and the absence of systematically adjudicated BARC bleeding data. Further multicenter studies with longer follow-up, standardized patient-reported outcome collection, systematic ischemia/viability and bleeding evaluation, detailed procedural reporting, and stronger control for treatment-selection bias are needed.

## Figures and Tables

**Table 1:** Baseline demographic and clinical characteristics according to treatment strategy.

Variable	CTO-PCI + OMT *n* = 153	OMT *n* = 98	*p*-Value
Age, years	66.26 ± 9.68	68.55 ± 9.68	0.069
Male sex	123/153 (80.4%)	75/98 (76.5%)	0.527
Female sex	30/153 (19.6%)	23/98 (23.5%)	0.527
Hypertension	151/153 (98.7%)	97/98 (99.0%)	1.000
Hypercholesterolemia/dyslipidemia	107/153 (69.9%)	79/98 (80.6%)	0.076
Non-insulin-dependent diabetes	38/153 (24.8%)	28/98 (28.6%)	0.558
Insulin-dependent diabetes	4/153 (2.6%)	4/98 (4.1%)	0.715
Previous stroke	11/153 (7.2%)	15/98 (15.3%)	0.055
Peripheral artery disease	10/153 (6.5%)	5/98 (5.1%)	0.788
Chronic kidney disease	26/153 (17.0%)	11/98 (11.2%)	0.274
COPD	10/153 (6.5%)	8/98 (8.2%)	0.625
Atrial fibrillation	26/153 (17.0%)	14/98 (14.3%)	0.601
**History of smoking**	53/153 (34.6%)	51/98 (52.0%)	**0.008**
Previous myocardial infarction	115/153 (75.2%)	63/98 (64.3%)	0.087
**Previous PCI**	124/153 (81.0%)	62/98 (63.3%)	**0.002**
Previous CABG	8/153 (5.2%)	12/98 (12.2%)	0.056

Values are presented as mean ± SD or *n*/*N* (%). *p*-values are nominal and were not adjusted for multiple comparisons. COPD, chronic obstructive pulmonary disease; PCI, percutaneous coronary intervention; CABG, coronary artery bypass grafting; OMT, optimal medical therapy. Bold *p*-values indicate nominal *p* < 0.05.

**Table 2:** Baseline angiographic characteristics according to treatment strategy.

Variable	CTO-PCI + OMT *n* = 153	OMT *n* = 98	Absolute Difference	*p*-Value
**Right coronary artery CTO**	85/153 (55.6%)	71/98 (72.4%)	−16.8 pp	**0.008**
Left anterior descending CTO	36/153 (23.5%)	15/98 (15.3%)	+8.2 pp	0.148
Left circumflex CTO	33/153 (21.6%)	12/98 (12.2%)	+9.4 pp	0.065
Left main CTO	0/153 (0.0%)	0/98 (0.0%)	0.0 pp	-
Difficult or very difficult CTO, J-CTO ≥ 2	84/153 (54.9%)	62/98 (63.3%)	−8.4 pp	0.535 *
Three-vessel coronary artery disease	100/153 (65.4%)	70/98 (71.4%)	−6.0 pp	0.336

Values are presented as *n*/*N* (%). Absolute difference represents CTO-PCI+OMT minus OMT and is expressed in percentage points. * *p*-value refers to the overall between-group comparison of J-CTO complexity. CTO, chronic total occlusion; J-CTO, Multicenter CTO Registry of Japan score; OMT, optimal medical therapy; PCI, percutaneous coronary intervention; pp, percentage points. Bold p-values indicate nominal *p* < 0.05.

**Table 3:** Procedural outcomes and periprocedural complications in the CTO-PCI + OMT group.

Procedural Outcome	*n*/*N*	%	95% CI
Technical success/successful CTO-PCI	129/153	84.3	77.7–89.2
Failed CTO-PCI	24/153	15.7	10.8–22.3
Favorable post-procedural TIMI flow	146/153	95.4	90.9–97.8
Favorable residual stenosis after PCI	147/153	96.1	91.7–98.2
Any recorded periprocedural complication	13/153	8.5	5.0–14.0
Procedural myocardial infarction	7/153	4.6	2.2–9.1
Coronary perforation	5/153	3.3	1.4–7.4
Cardiac tamponade	2/153	1.3	0.4–4.6
Stroke	1/153	0.7	0.1–3.6
Periprocedural death	0/153	0.0	0.0–2.4
Urgent CABG	0/153	0.0	0.0–2.4

Values are shown as *n*/*N* and percentage. Confidence intervals were calculated using the Wilson method. Technical success was defined as successful recanalization of the target CTO with restoration of TIMI grade 3 antegrade flow and residual stenosis <30% in the treated segment. Favorable post-procedural TIMI flow and favorable residual stenosis after PCI were recorded as component procedural fields and interpreted descriptively. Any recorded periprocedural complication refers to the occurrence of at least one of the listed complications; therefore, individual complication counts may exceed the number of patients with any complication. CABG, coronary artery bypass grafting; CTO-PCI, chronic total occlusion percutaneous coronary intervention; TIMI, Thrombolysis in Myocardial Infarction.

**Table 4:** Baseline and 12-month medical therapy according to treatment strategy.

Medication Class	CTO-PCI + OMT Baseline *n* = 153	OMT Baseline *n* = 98	*p*-Value	CTO-PCI + OMT 12 Months *n* = 135	OMT 12 Months *n* = 81	*p*-Value
Aspirin/ASA	**147/153 (96.1%)**	**84/98 (85.7%)**	**0.004**	114/135 (84.4%)	64/81 (79.0%)	0.357
P2Y12 receptor inhibitor	**150/153 (98.0%)**	**77/98 (78.6%)**	**<0.001**	**115/135 (85.2%)**	**50/81 (61.7%)**	**<0.001**
Beta-blocker	111/153 (72.5%)	72/98 (73.5%)	1.000	89/135 (65.9%)	52/81 (64.2%)	0.883
ACE inhibitor	90/153 (58.8%)	65/98 (66.3%)	0.287	77/135 (57.0%)	51/81 (63.0%)	0.475
Angiotensin II receptor blocker	30/153 (19.6%)	19/98 (19.4%)	1.000	27/135 (20.0%)	22/81 (27.2%)	0.243
Calcium-channel blocker	67/153 (43.8%)	46/98 (46.9%)	0.697	67/135 (49.6%)	45/81 (55.6%)	0.482
Statin	147/153 (96.1%)	89/98 (90.8%)	0.104	114/135 (84.4%)	66/81 (81.5%)	0.577
Other lipid-lowering drug	19/153 (12.4%)	10/98 (10.2%)	0.688	27/135 (20.0%)	14/81 (17.3%)	0.721
Nitrate	**45/153 (29.4%)**	**46/98 (46.9%)**	**0.007**	**39/135 (28.9%)**	**43/81 (53.1%)**	**<0.001**
Trimetazidine	40/153 (26.1%)	37/98 (37.8%)	0.068	40/135 (29.6%)	33/81 (40.7%)	0.104
Ranolazine	0/153 (0.0%)	2/98 (2.0%)	0.151	5/135 (3.7%)	8/81 (9.9%)	0.079
Ivabradine	5/153 (3.3%)	1/98 (1.0%)	0.409	15/135 (11.1%)	8/81 (9.9%)	0.824
Antianginal medication classes, mean ± SD	**1.75 ± 1.01**	**2.08 ± 1.00**	**0.012**	**1.89 ± 1.12**	**2.33 ± 1.19**	**0.007**
Secondary-prevention medication classes, mean ± SD	**3.81 ± 0.62**	**3.51 ± 0.92**	**0.005**	**3.51 ± 0.79**	**3.30 ± 0.86**	0.069

Values are presented as *n*/*N* (%) unless otherwise indicated. Twelve-month medication analyses were restricted to patients with available follow-up medication data. Antianginal medication classes included beta-blockers, calcium-channel blockers, nitrates, trimetazidine, ranolazine, and ivabradine. Secondary-prevention medication classes included aspirin, P2Y12 receptor inhibitors, ACE inhibitors, angiotensin II receptor blockers, statins, and other lipid-lowering drugs. *p*-values are nominal and were not adjusted for multiple comparisons. ACE, angiotensin-converting enzyme; CTO-PCI, chronic total occlusion percutaneous coronary intervention; OMT, optimal medical therapy. Bold *p*-values and corresponding highlighted values indicate nominal *p* < 0.05.

**Table 5:** Seattle Angina Questionnaire domain scores at baseline and 12-month follow-up.

SAQ Domain	CTO-PCI + OMT Baseline	CTO-PCI + OMT 12 Months	CTO-PCI + OMT Δ	OMT Baseline	OMT 12 Months	OMT Δ	Between-Group Δ Difference	*p*-Value for Δ
Physical Limitation	67.97 ± 17.09	72.95 ± 15.46	**+4.98 ± 11.03**	61.16 ± 20.30	67.51 ± 16.48	**+6.35 ± 12.48**	−1.37 points; 95% CI, −4.69 to 1.94	0.415
Angina Stability	54.23 ± 26.45	68.93 ± 23.47	**+14.71 ± 30.35**	54.63 ± 26.84	66.67 ± 16.30	**+12.04 ± 27.42**	+2.67 points; 95% CI, −5.24 to 10.57	0.506
Angina Frequency	70.81 ± 17.97	92.35 ± 12.37	**+21.54 ± 18.37**	63.09 ± 20.10	83.09 ± 15.14	**+20.00 ± 19.36**	+1.54 points; 95% CI, −3.72 to 6.81	0.563
Treatment Satisfaction	69.25 ± 17.24	76.12 ± 16.02	**+6.87 ± 15.91**	63.27 ± 22.97	74.38 ± 17.42	**+11.11 ± 14.35**	−4.24 points; 95% CI, −8.38 to −0.10	**0.045**
Quality of Life	62.69 ± 24.88	74.54 ± 18.96	**+11.85 ± 18.83**	61.70 ± 25.44	74.25 ± 15.43	**+12.55 ± 17.03**	−0.71 points; 95% CI, −5.61 to 4.20	0.777

Values are presented as mean ± SD unless otherwise indicated. A was calculated as the 12-month score minus the baseline score. Positive values indicate improvement. The SAQ complete-case population included patients with complete baseline and 12-month data for all five SAQ domains: CTO-PCI + OMT, *n* = 136; OMT, *n* = 81. Between-group A difference represents CTO-PCI + OMT minus OMT. Because baseline differences were present in some SAQ domains, unadjusted follow-up comparisons should be interpreted descriptively; baseline-adjusted analyses are presented separately. *p*-values are nominal and were not adjusted for multiple comparisons across SAQ domains. SAQ, Seattle Angina Questionnaire; OMT, optimal medical therapy; PCI, percutaneous coronary intervention; CI, confidence interval. Bold *p*-values indicate nominal *p* < 0.05.

**Table 6:** Adjusted models for SAQ Angina Frequency outcomes. Table note: Linear regression was adjusted for treatment strategy, baseline SAQ Angina Frequency, age, sex, baseline LVEF, history of smoking, previous PCI, and J-CTO score. The extended logistic model included the same covariates. Clinically relevant improvement was defined as an increase of at least 10 points in SAQ Angina Frequency from baseline to 12 months. SAQ AF, Seattle Angina Questionnaire Angina Frequency; OR, odds ratio; CI, confidence interval.

Model	Analytic Population	Outcome	Treatment-Effect Estimate for CTO-PCI + OMT vs. OMT	95% CI	*p*-Value
Multivariable ANCOVA	Complete-case SAQ population	12-month SAQ Angina Frequency	β = 7.07	3.36–10.79	<0.001
Propensity-score overlap-weighted ANCOVA	Complete-case SAQ population	12-month SAQ Angina Frequency	β = 6.55	2.39–10.70	0.002
Propensity-score overlap-weighted change-score analysis	Complete-case SAQ population	ΔSAQ Angina Frequency	β = 6.39	0.53–12.24	0.033
Baseline-adjusted logistic regression	Complete-case SAQ population	ΔSAQ Angina Frequency ≥ 10 points	OR = 6.03	2.34–15.48	<0.001
Extended multivariable logistic regression	Complete-case SAQ population	ΔSAQ Angina Frequency ≥ 10 points	OR = 6.46	2.34–17.87	<0.001
Propensity-score overlap-weighted logistic regression	Complete-case SAQ population	ΔSAQ Angina Frequency ≥ 10 points	OR = 6.61	2.48–17.62	<0.001
Conservative full-cohort responder sensitivity analysis	Full cohort	ΔSAQ Angina Frequency ≥ 10 points; missing follow-up classified as no improvement	OR = 3.70	1.79–7.63	<0.001

**Table 7:** Secondary clinical outcomes at 12-month follow-up.

Outcome	CTO-PCI + OMT	OMT	Effect Estimate/ Absolute Difference	95% CI	*p*-Value
**CCS angina class at 12 months**					
CCS 0	84/136 (61.8%)	39/81 (48.1%)	Absolute difference +13.7 pp	—	0.065
CCS I	17/136 (12.5%)	19/81 (23.5%)	Absolute difference −11.0 pp	—	0.040
CCS II	22/136 (16.2%)	16/81 (19.8%)	Absolute difference −3.6 pp	—	0.580
CCS III	9/136 (6.6%)	6/81 (7.4%)	Absolute difference −0.8 pp	—	0.790
CCS IV	4/136 (2.9%)	1/81 (1.2%)	Absolute difference +1.7 pp	—	0.653
Improvement ≥1 CCS class	115/136 (84.6%)	72/81 (88.9%)	Absolute difference −4.3 pp	—	NS
Improvement ≥2 CCS classes	96/136 (70.6%)	56/81 (69.1%)	Absolute difference +1.5 pp	—	NS
**Left ventricular ejection fraction**					
**Baseline LVEF, %**	49.20 ± 10.52	46.00 ± 11.85	Mean difference +3.20 pp	—	0.030
**12-month LVEF, %**	50.19 ± 8.84	46.77 ± 9.63	Mean difference +3.42 pp	—	0.006
**LVEF change in paired subgroup,** **percentage points**	+0.26 ± 3.03	−0.88 ± 3.68	Mean difference +1.14 pp	—	0.020
**Clinical follow-up outcomes**					
**Cumulative cardiovascular** **rehospitalization at 12 months**	11/153 (7.2%)	19/98 (19.4%)	OR = 0.32	0.15–0.71	0.005
Deaths during follow-up	12/153	12/98	24 deaths total	—	—
12-month overall survival estimate	92.0%	87.4%	Difference +4.6 pp	—	0.225

Values are presented as mean ± SD, *n*/*N* (%), or percentage. Effect estimates and absolute differences are presented before *p*-values to emphasize magnitude and precision. For categorical outcomes, absolute difference represents CTO-PCI + OMT minus OMT and is expressed in percentage points. Rehospitalization was analyzed as a cumulative 12-month binary outcome. LVEF was analyzed as a secondary exploratory outcome. Survival analyses were exploratory because only 24 deaths occurred during follow-up. *p*-values are nominal and were not adjusted for multiple comparisons. CCS, Canadian Cardiovascular Society; LVEF, left ventricular ejection fraction; OR, odds ratio; CI, confidence interval; OMT, optimal medical therapy; PCI, percutaneous coronary intervention; pp, percentage points; NS, not statistically significant.

## Data Availability

The data presented in this study are available from the corresponding author upon reasonable request. The data are not publicly available due to privacy and ethical restrictions related to patient-level clinical information. Access to anonymized data may be considered following approval by the relevant institutional ethics committee.

## Supplementary Materials

The following supporting information can be downloaded at: https://www.mdpi.com/article/10.3390/jcm15145668/s1. Table S1: Documented ischemia and viability assessment according to treatment strategy; Table S2: Baseline covariate balance before and after propensity-score overlap weighting; Table S3: Baseline characteristics of patients with complete versus missing SAQ follow-up; Table S4: Baseline-adjusted analysis of 12-month SAQ domain scores; Table S5: Clinically meaningful improvement across SAQ domains; Table S6: Logistic regression models for cumulative 12-month cardiovascular rehospitalization.

## Abbreviations

Abbreviation	Definition
ACE	Angiotensin-converting enzyme
ANCOVA	Analysis of covariance
ARB	Angiotensin II receptor blocker
ASA	Acetylsalicylic acid
CABG	Coronary artery bypass grafting
CCS	Canadian Cardiovascular Society
CI	Confidence interval
COPD	Chronic obstructive pulmonary disease
CTO	Chronic total occlusion
CTO-PCI	Percutaneous coronary intervention for chronic total occlusion
ESS	Effective sample size
IPTW	Inverse probability of treatment weighting
J-CTO	Multicenter CTO Registry of Japan score
LAD	Left anterior descending artery
LCX	Left circumflex artery
LVEF	Left ventricular ejection fraction
MACCE	Major adverse cardiac and cerebrovascular event
OMT	Optimal medical therapy
OR	Odds ratio
PCI	Percutaneous coronary intervention
pp	Percentage points
PS	Propensity score
QoL	Quality of life
RCA	Right coronary artery
SAQ	Seattle Angina Questionnaire
SAQ AF	Seattle Angina Questionnaire Angina Frequency domain
SD	Standard deviation
SMD	Standardized mean difference
TIMI	Thrombolysis in Myocardial Infarction

## References

[1] Fefer P., Knudtson M.L., Cheema A.N., Galbraith P.D., Osherov A.B., Yalonetsky S., Gannot S., Samuel M., Weisbrod M., Bierstone D. (2012). Current perspectives on coronary chronic total occlusions: The Canadian Multicenter Chronic Total Occlusions Registry. J. Am. Coll. Cardiol..

[2] Xenogiannis I., Pavlidis A.N., Karamasis G.V. (2024). Editorial: Contemporary percutaneous interventions for coronary chronic total occlusions. Front. Cardiovasc. Med..

[3] Synetos A., Koliastasis L., Ktenopoulos N., Katsaros O., Vlasopoulou K., Drakopoulou M., Apostolos A., Tsalamandris S., Latsios G., Toutouzas K. (2025). Recent Advances in Coronary Chronic Total Occlusions. J. Clin. Med..

[4] Vrints C., Andreotti F., Koskinas K.C., Rossello X., Adamo M., Ainslie J., Banning A.P., Budaj A., Buechel R.R., Chiariello G.A. (2024). 2024 ESC Guidelines for the management of chronic coronary syndromes. Eur. Heart J..

[5] Lee S.W., Lee P.H., Ahn J.M., Park D.W., Yun S.C., Han S., Kang H., Kang S.J., Kim Y.H., Lee C.W. (2019). Randomized Trial Evaluating Percutaneous Coronary Intervention for the Treatment of Chronic Total Occlusion. Circulation.

[6] Werner G.S., Martin-Yuste V., Hildick-Smith D., Boudou N., Sianos G., Gelev V., Rumoroso J.R., Erglis A., Christiansen E.H., Escaned J. (2018). A randomized multicentre trial to compare revascularization with optimal medical therapy for the treatment of chronic total coronary occlusions. Eur. Heart J..

[7] Obedinskiy A.A., Kretov E.I., Boukhris M., Kurbatov V., Osiev A.G., Ibn Elhadj Z., Obedinskaya N.R., Kasbaoui S., Grazhdankin I.O., Prokhorikhin A.A. (2018). The IMPACTOR-CTO Trial. JACC Cardiovasc. Interv..

[8] Macherey-Meyer S., Salem K., Heyne S., Meertens M.M., Finke K., Mauri V., Baldus S., Adler C., Lee S. (2024). Percutaneous Coronary Intervention versus Optimal Medical Therapy in Patients with Chronic Total Occlusion: A Meta-Analysis. J. Clin. Med..

[9] Coerkamp C.F., van Veelen A., Mashayekhi K.A., Stojkovic S.M., Juricic S.A., Claessen B.E.P.M., van der Schaaf R.J., Hoebers L.P.C., Elias J., Henriques J.P.S. (2024). Long-Term Survival and Angina After Chronic Total Occlusion Percutaneous Coronary Intervention Compared with Medical Therapy: A Meta-Analysis. J. Am. Heart Assoc..

[10] Werner G.S., Kim J.-H., Hildick-Smith D., Kang D.-Y., Martin Yuste V., Ahn J.-M., Boudou N., Park D.-W., Louvard Y., Christiansen E.H. (2026). Quality of Life After Percutaneous Coronary Intervention or Medical Therapy for Chronic Total Coronary Occlusions: EUROCTO and DECISION-CTO Meta-Analysis. J. Am. Coll. Cardiol..

[11] Khan S., Sajjad U., Fawaz S., Butt H., Simpson R., Ibrahim A., Robertson C., Kelly P., Mohdnazri S.R., Tang K. (2026). A Randomized, Placebo-Controlled Trial of Chronic Total Occlusion Percutaneous Coronary Intervention in Stable Angina: ORBITA-CTO. J. Am. Coll. Cardiol.

[12] U.S. Food and Drug Administration (2009). Patient-Reported Outcome Measures: Use in Medical Product Development to Support Labeling Claims: Guidance for Industry.

[13] Calvert M., Blazeby J., Altman D.G., Revicki D.A., Moher D., Brundage M.D., CONSORT PRO Group (2013). Reporting of patient-reported outcomes in randomized trials: The CONSORT PRO extension. JAMA.

[14] Spertus J.A., Winder J.A., Dewhurst T.A., Deyo R.A., Prodzinski J., McDonell M., Fihn S.D. (1995). Development and evaluation of the Seattle Angina Questionnaire: A new functional status measure for coronary artery disease. J. Am. Coll. Cardiol..

[15] Weintraub W.S., Spertus J.A., Kolm P., Maron D.J., Zhang Z., Jurkovitz C., Zhang W., Hartigan P.M., Lewis C., Veledar E. (2008). Effect of PCI on quality of life in patients with stable coronary disease. N. Engl. J. Med..

[16] Spertus J.A., Jones P.G., Maron D.J., O’Brien S.M., Reynolds H.R., Rosenberg Y., Stone G.W., Harrell F.E., Boden W.E., Weintraub W.S. (2020). Health-status outcomes with invasive or conservative care in coronary disease. N. Engl. J. Med..

