# Supplementary material for: Patient-Reported Health Status and Clinical Outcomes After CTO-PCI Versus Optimal Medical Therapy: A 12-Month Retrospective Observational Cohort Analysis
Source: J Clin Med. 2026 Jul 20;15(14):5668. doi: 10.3390/jcm15145668 (PMC13412261; doi:10.3390/jcm15145668)
Supplement: Supplementary file 1 [file jcm-15-05668-s001.zip › jcm-4400633-supplementary.pdf]

Supplementary tables:

Supplementary Table S1. Baseline covariate balance before and after propensity-score overlap weighting.

Covariate	Absolute SMD before weighting	Absolute SMD after overlap weighting
Age	0.237	0.000
Male sex	0.094	0.000
Hypertension	0.027	0.000
Dyslipidemia	0.248	0.000
Diabetes mellitus	0.113	0.000
Previous stroke	0.258	0.000
Peripheral artery disease	0.061	0.000
Chronic kidney disease	0.166	0.000
COPD	0.062	0.000
Atrial fibrillation	0.074	0.000
History of smoking	0.355	0.000
Previous myocardial infarction	0.237	0.000
Previous PCI	0.403	0.000
Previous CABG	0.249	0.000
Baseline LVEF	0.286	0.000
Baseline CCS class	0.044	0.000
Baseline SAQ Physical Limitation	0.368	0.000
Baseline SAQ Angina Stability	0.014	0.000
Baseline SAQ Angina Frequency	0.451	0.000
Baseline SAQ Treatment Satisfaction	0.399	0.000
Baseline SAQ Quality of Life	0.006	0.000
RCA CTO	0.356	0.000
LAD CTO	0.208	0.000
LCX CTO	0.250	0.033
J-CTO score	0.161	0.000
Three-vessel coronary artery disease	0.130	0.000
Dobutamine stress echocardiography	0.196	0.000
Baseline antianginal medication count	0.327	0.000
Baseline secondary-prevention medication count	0.383	0.000

Values are absolute standardized mean differences. Propensity scores were estimated using baseline demographic, clinical, echocardiographic, angiographic, patient-reported, and treatment-intensity variables. Overlap weights were applied to improve measured covariate balance between the CTO-PCI + OMT and OMT groups. LCX CTO was assessed for balance but was not included in the final propensity-score model. SMD, standardized mean difference; CTO, chronic total occlusion; PCI, percutaneous coronary intervention; OMT, optimal medical therapy; LVEF, left ventricular ejection fraction; CCS, Canadian Cardiovascular Society; SAQ, Seattle Angina Questionnaire; RCA, right coronary artery; LAD, left anterior descending artery; LCX, left circumflex artery.

Supplementary Table S2. Documented ischemia and viability assessment according to treatment strategy.

Assessment variable	CTO-PCI + OMT n = 153	OMT n = 98	Total n = 251	p-value
Dobutamine stress echocardiography performed	**51/153 (33.3%)**	**42/98 (42.9%)**	**93/251 (37.1%)**	**0.142**
Quantitative ischemic burden	Not uniformly available	Not uniformly available	Not uniformly available	—
Quantitative viability extent	Not uniformly available	Not uniformly available	Not uniformly available	—
Stress CMR / nuclear perfusion / PET data	Not systematically captured	Not systematically captured	Not systematically captured	—

Values are presented as n/N (%). The p-value for dobutamine stress echocardiography was calculated using Fisher's exact test. Dobutamine stress echocardiography was the functional ischemia/viability testing modality systematically captured in the available dataset. Quantitative ischemic burden, quantitative viability extent, stress cardiac magnetic resonance, nuclear perfusion imaging, and positron-emission tomography data were not uniformly available. CMR, cardiac magnetic resonance; PET, positron-emission tomography; CTO-PCI, chronic total occlusion percutaneous coronary intervention; OMT, optimal medical therapy.

Supplementary Table S3. Baseline characteristics of patients with complete versus missing SAQ follow-up.

Variable	Complete SAQ follow-up *n* = 217	Missing SAQ follow-up *n* = 34	*p*-value
CTO-PCI + OMT treatment strategy	136/217 (62.7%)	17/34 (50.0%)	0.187
**Age, years**	**66.44 ± 9.50**	**71.74 ± 10.03**	**0.006**
Male sex	175/217 (80.6%)	23/34 (67.6%)	0.111
Hypertension	214/217 (98.6%)	34/34 (100.0%)	1.000
Dyslipidemia	163/217 (75.1%)	23/34 (67.6%)	0.400
Diabetes mellitus	60/217 (27.6%)	14/34 (41.2%)	0.155
Previous stroke	23/217 (10.6%)	3/34 (8.8%)	1.000
Peripheral artery disease	12/217 (5.5%)	3/34 (8.8%)	0.436
Chronic kidney disease	31/217 (14.3%)	6/34 (17.6%)	0.605
COPD	17/217 (7.8%)	1/34 (2.9%)	0.481
**Atrial fibrillation**	**29/217 (13.4%)**	**11/34 (32.4%)**	**0.010**
History of smoking	91/217 (41.9%)	13/34 (38.2%)	0.713
Previous myocardial infarction	155/217 (71.4%)	23/34 (67.6%)	0.686
Previous PCI	159/217 (73.3%)	27/34 (79.4%)	0.532
Previous CABG	16/217 (7.4%)	4/34 (11.8%)	0.326
**Baseline LVEF, %**	**49.07 ± 10.34**	**40.79 ± 13.41**	**0.001**
Baseline CCS class	2.85 ± 0.74	2.97 ± 0.63	0.325
Baseline CCS III/IV	145/217 (66.8%)	27/34 (79.4%)	0.167
Baseline SAQ Angina Frequency	67.93 ± 19.12	66.76 ± 17.53	0.724
RCA CTO	132/217 (60.8%)	24/34 (70.6%)	0.343
J-CTO score	1.76 ± 1.10	1.62 ± 0.92	0.418
Three-vessel coronary artery disease	144/217 (66.4%)	26/34 (76.5%)	0.324
Death before 12-month SAQ reassessment	0/217 (0.0%)	24/34 (70.6%)	—
Lost to SAQ follow-up	0/217 (0.0%)	10/34 (29.4%)	—

Values are presented as mean ± *SD* or *n/N* (%). The complete-case SAQ population included patients with available baseline and 12-month SAQ data for all five SAQ domains. Missing SAQ follow-up was due to death before 12-month SAQ reassessment or loss to SAQ follow-up. p-values are descriptive and compare patients with complete versus missing SAQ follow-up. SAQ, Seattle Angina Questionnaire; CTO, chronic total occlusion; PCI, percutaneous coronary intervention; OMT, optimal medical therapy; LVEF, left ventricular ejection fraction; CCS, Canadian Cardiovascular Society; CABG, coronary artery bypass grafting; COPD, chronic obstructive pulmonary disease; RCA, right coronary artery. Bold p-values and corresponding highlighted values indicate nominal $p < 0.05$.

Supplementary Table S4. Baseline-adjusted analysis of 12-month SAQ domain scores.

SAQ domain	Baseline-adjusted treatment effect for CTO-PCI + OMT vs. OMT	95% CI	p-value
Physical Limitation	β = 0.85	−1.94 to 3.65	0.548
Angina Stability	β = 2.35	−3.29 to 7.99	0.413
**Angina Frequency**	**β = 7.27**	**3.72 to 10.82**	**<0.001**
Treatment Satisfaction	β = −1.55	−5.08 to 1.97	0.387
Quality of Life	β = −0.20	−3.76 to 3.37	0.914

Each model included treatment strategy and the corresponding baseline SAQ domain score, with the 12-month SAQ domain score as the dependent variable. Positive β values favor CTO-PCI + OMT. p-values are nominal and were not adjusted for multiple comparisons. SAQ, Seattle Angina Questionnaire; CTO-PCI, chronic total occlusion percutaneous coronary intervention; OMT, optimal medical therapy; CI, confidence interval. Bold p-values indicate nominal $p < 0.05$.

Supplementary Table S5. Clinically meaningful improvement across SAQ domains.

SAQ domain	≥5-point improvement CTO-PCI + OMT	≥5-point improvement OMT	≥10-point improvement CTO-PCI + OMT	≥10-point improvement OMT	≥20-point improvement CTO-PCI + OMT	≥20-point improvement OMT
Physical Limitation	54/136 (39.7%)	35/81 (43.2%)	31/136 (22.8%)	26/81 (32.1%)	13/136 (9.6%)	8/81 (9.9%)
Angina Stability	77/136 (56.6%)	39/81 (48.1%)	77/136 (56.6%)	39/81 (48.1%)	77/136 (56.6%)	39/81 (48.1%)
**Angina Frequency**	**116/136 (85.3%)**	**59/81 (72.8%)**	**116/136 (85.3%)**	**59/81 (72.8%)**	**92/136 (67.6%)**	**46/81 (56.8%)**
Treatment Satisfaction	85/136 (62.5%)	56/81 (69.1%)	54/136 (39.7%)	45/81 (55.6%)	16/136 (11.8%)	14/81 (17.3%)
Quality of Life	86/136 (63.2%)	52/81 (64.2%)	48/136 (35.3%)	38/81 (46.9%)	37/136 (27.2%)	20/81 (24.7%)

Clinically meaningful improvement was defined descriptively using thresholds of ≥5, ≥10, and ≥20 points from baseline to 12 months. Because several SAQ domains have discrete scoring intervals, some thresholds may yield identical counts. These analyses were exploratory and were not adjusted for multiple comparisons. SAQ, Seattle Angina Questionnaire; OMT, optimal medical therapy; PCI, percutaneous coronary intervention.

Supplementary Table S6. Logistic regression models for cumulative 12-month cardiovascular rehospitalization.

Model	Effect estimate for CTO-PCI + OMT vs. OMT	95% CI	p-value
Unadjusted logistic regression	**OR = 0.32**	**0.15–0.71**	**0.005**
Baseline SAQ QoL-adjusted logistic regression	**OR = 0.31**	**0.14–0.69**	**0.004**
Extended multivariable logistic regression	**OR = 0.31**	**0.13–0.76**	**0.011**
Propensity-score overlap-weighted logistic regression	**OR = 0.33**	**0.13–0.86**	**0.023**

Cardiovascular rehospitalization was analyzed as a cumulative 12-month binary outcome because exact dates of rehospitalization were not consistently available for all patients. The extended multivariable model was adjusted for baseline SAQ Quality of Life, age, sex, baseline LVEF, smoking history, previous PCI, and J-CTO score. The propensity-score overlap-weighted model used robust standard errors. All models were exploratory and should be interpreted as adjusted associations rather than causal treatment-effect estimates. SAQ QoL, Seattle Angina Questionnaire Quality of Life; OR, odds ratio; CI, confidence interval; OMT, optimal medical therapy; PCI, percutaneous coronary intervention; LVEF, left ventricular ejection fraction; J-CTO, Multicenter CTO Registry of Japan score.
